# NOTCH4 maintains quiescent mesenchymal-like breast cancer stem cells via transcriptionally activating SLUG and GAS1 in triple-negative breast cancer

**DOI:** 10.7150/thno.38875

**Published:** 2020-01-19

**Authors:** Lei Zhou, Dong Wang, Dandan Sheng, Jiahui Xu, Weilong Chen, Yuanyuan Qin, Ruikai Du, Xiaoli Yang, Xueyan He, Ni Xie, Suling Liu, Lixing Zhang

**Affiliations:** 1 Fudan University Shanghai Cancer Center & Institutes of Biomedical Sciences; Shanghai Medical College; Key Laboratory of Breast Cancer in Shanghai; Innovation Center for Cell Signaling Network: Cancer Institute, Fudan University, Shanghai 200032, China;; 2 The CAS Key Laboratory of Innate Immunity and Chronic Disease, School of Life Sciences, University of Science & Technology of China, Hefei, Anhui 230027, China;; 3 WPI Nano Life Science Institute (WPI-NanoLSI), Kanazawa University, Kakuma-machi, Kanazawa 920-1192, Japan;; 4 State Key Laboratory of Space Medicine Fundamentals and Application, China Astronaut Research and Training Center, Beijing 100193, China; 5 Shenzhen Second People's Hospital, First Affiliated Hospital of Shenzhen University, Shenzhen University, Shenzhen 518035, China;

**Keywords:** NOTCH4, SLUG, GAS1, mesenchymal-like breast cancer stem cell (ML-BCSC), TNBC

## Abstract

**Rationale:** NOTCH4 receptor has been implicated in triple-negative breast cancer (TNBC) development and breast cancer stem cell (BCSC) regulation. However, the potential of NOTCH4 as a BCSC marker and the underlying mechanisms remain unclear.

**Methods:** In this study, we determined the expression and activation of NOTCH4 in breast cancer cell lines and tumor samples by qRT-PCR, western blotting and immunohistochemistry. Subsequently, *in vitro* and *in vivo* serial dilution experiments were performed to demonstrate the application of NOTCH4 as an efficient mesenchymal-like (ML)-BCSC marker in TNBC. Stable overexpression of activated NOTCH4 and knockdown cell lines were established using lentivirus. RNA-seq and qRT-PCR were employed to reveal the downstream effectors of NOTCH4, followed by dual-luciferase reporter and chromatin immunoprecipitation assays to identify the genuine binding sites of NOTCH4 on SLUG and GAS1 promoters. Transwell assay, mammosphere formation and chemoresistance experiments were performed to determine the effects of SLUG, GAS1 and NOTCH4 on the mesenchymal-like characteristics of TNBC cells. Survival analysis was used to study the relation of NOTCH4, SLUG and GAS1 with prognosis of breast cancer.

**Results:** NOTCH4 is aberrantly highly expressed and activated in TNBC, which contributes to the maintenance of ML-BCSCs. Furthermore, NOTCH4 shows significantly higher efficiency in labeling ML-BCSCs than the currently commonly used CD24^-^CD44^+^ marker. Mechanistically, NOTCH4 transcriptionally upregulates SLUG and GAS1 to promote EMT and quiescence in TNBC, respectively. The effects of NOTCH4 can be mimicked by simultaneous overexpression of SLUG and GAS1. Moreover, SLUG is also involved in harnessing GAS1, a known tumor suppressor gene, via its anti-apoptotic function.

**Conclusions:** Our findings reveal that the NOTCH4-SLUG-GAS1 circuit serves as a potential target for tumor intervention by overcoming stemness of ML-BCSCs and by conquering the lethal chemoresistance and metastasis of TNBC.

## Introduction

Breast cancer is the most common and second death-leading cancer type in women 1. Transcriptome profiling according to the intrinsic molecular portraits of breast cancer has identified several major subcategories, such as Luminal, HER2^+^ and Triple-negative 2, of which Triple-negative Breast Cancer (TNBC) is a heterogenous and refractory subtype, featured by enhanced chemoresistance, relapse, and metastasis 3, 4. The malignant features of TNBC is proposed to be mediated by breast cancer stem cells (BCSCs) 5-7, for which the current commonly used markers are CD24^-^CD44^+^ 8-10 and aldehyde dehydrogenase (ALDH) 11-13, with CD24^-^CD44^+^ labeling the Mesenchymal-like CSCs and ALDH^+^ defining the Epithelial-like counterpart 14. However, our recent work showed that TNBC cells intrinsically express high level of CD44, with almost the whole cell population defined as CD24^-^CD44^+^ upon flow cytometry analysis 15, indicating the inefficiency of CD24^-^CD44^+^ as a BCSC marker in TNBC. Therefore, novel markers are in desire to more efficiently and accurately enrich TNBC BCSCs, in benefit of precisely targeting BCSCs for prognostic and therapeutic purposes.

NOTCH pathway, highly conserved through evolution and involved in regulating stem cell maintenance and cell fate control, is frequently dysregulated and implicated in cancer occurrence, chemoresistance, relapse, and metastasis 16-19. Previous studies have demonstrated that NOTCH receptors can function as either oncogene or tumor suppressor gene via divergent downstream signaling pathways 20, depending on the cellular context 21. Among all four NOTCH receptors, NOTCH4 is more attractive in TNBC for the following reasons: (1) it was initially identified in virus triggered mouse mammary tumor (initially designated as int-3) and thus directly linked to tumor initiation 22; (2) NOTCH4 has been reported to be implicated in cancer progression 23 and BCSC regulation 24, and function as a melanoma stem cell marker 25; (3) NOTCH4 has been recently shown to play a role in TNBC, as two independent groups observed high expression of NOTCH4 in TNBC 26, 27 to highlight its clinical significance. However, whether NOTCH4 could be used as a BCSC marker in TNBC and what are the underlying mechanisms have not been clearly answered yet.

In the present study, we reported that NOTCH4 could be used to enrich ML-BCSCs in TNBC. The NOTCH4^+^ BCSCs were highly potential in tumor initiation and were characterized by enhanced invasion and chemoresistance ability. Mechanistically, NOTCH4 induced EMT via direct upregulation of SLUG, a master regulator of EMT in both normal and CSCs, and caused quiescence by transcriptionally activating GAS1, a well-known cell cycle arrest regulator 28 and Hedgehog pathway potentiator 29. Subsequently, SLUG and GAS1 worked together to facilitate the maintenance of the ML-BCSC portraits. We further demonstrated that interference of SLUG and GAS1 could destruct the malignant phenotypes induced by NOTCH4. Our results provided a pre-clinical promise for TNBC treatment to overcome the metastasis and chemoresistance by targeting the NOTCH4-SLUG-GAS1 circuit.

## Materials and Methods

### Ethics statement

All paraffin sections used in this study were obtained from Shanghai Cancer Center (Shanghai, China). Written informed consent was obtained from all patients before surgery, as advocated by the regional ethics committee. The study was approved by Fudan University Shanghai Cancer Center Institutional Review Board (FUSCC 050432-4-1212B). All mouse experiments were conducted in accordance with standard operating procedures approved by the University Committee on the Use and Care of Animals at University of Science and Technology of China (USTCACUC1401020).

### Cell culture and tumor transplantation

Breast cancer cell lines SUM149 and SUM159 were purchased from Asterland Bioscience, cultured in Han's F12 medium supplemented with 5% FBS (Ausbian, Sydney, Australia) and 1% streptomycin/penicillin, 1 mg/mL hydrocortisone, and 5 mg/mL insulin (Beyotime, Shanghai, China). MDA-MB-231, MDA-MB-436, T47D, MCF-7, BT474, SK-BR-3 were purchased from ATCC and were cultured according to ATCC recommendations. The cells were maintained in a 37 °C incubator with 5% carbon dioxide (CO_2_). Cell identity was monitored by STR profiling and mycoplasma contamination was routinely monitored by PCR. All mice were housed in AAALAC-accredited specific pathogen-free rodent facilities at University of Science and Technology of China. 4-5 weeks old female nude mice were purchased from Charles River (Beijing, China), housed in sterilized and ventilated microisolator cages, and supplied with autoclaved commercial chow and sterile water. Tumorigenicity was conducted by injecting 1 million of MDA-MB-231 cells or SUM149 cells, which were suspended in FBS and mixed with Matrigel at equal volume, into the fourth mammary fat pads of mice, with 3-4 replicates in each group. Tumor occurrence and growth were monitored and/or measured once a week. Tumor was considered as a spheroid and the volume was calculated as V=4π/3× (Length/2) × (Width/2)^2^.

### Plasmid construction

cDNA of NOTCH4 Intracellular Domain (NICD4) was synthesized at Sangon Biotech (Shanghai, China) and cloned into pSIN-EF1α-IRES-puro vector using OneStep kit (#C112, Vazyme, Nanjing, China). FLAG tag was introduced at the C-terminus of NICD4 to facilitate ChIP experiment. SLUG and GAS1 coding sequences were amplified from cDNA of NICD4-overexpressing SUM149 using the indicated primer pairs BamHI-SLUG-Fd & EcoRI-SLUG-Rv and BamHI-GAS1-Fd & EcoRI-GAS1-Rv, followed by seamless cloning into pSIN-EF1α-IRES-NEO or pSIN-EF1α-IRES-BSD vector. The primers were listed in **Table S1.** shRNA vectors used in this study were constructed following our previously reported SuperSH method 15 and the shRNA sequences were obtained from the TRC project. The pLKO.1-puro vector was purchased from Addgene (#8453). The primers used for shRNA vector construction were attached in **Table S2**.

### Lentiviral production and infection

The lentivirus vectors were co-transfected with two packaging vectors psPAX2 (#12260, Addgene, Watertown, MA) and pMD2.G (#12259, Addgene) at a ratio of 2 μg: 1 μg: 1 ug into packaging cell HEK293T using 12 μg Polyethylenimine (Sigma, MA, USA) in 6 well cell culture plate, followed by 48 h incubation before harvesting the supernatant containing virus. Target cells were infected with the filtered lentivirus-containing supernatant in the presence of polybrene (Sigma) for 24 h and selected with 2 μg/mL puromycin (Sigma) or 5 μg/mL blasticidin (Gibco, NY, USA) for at least 7 days to establish stable cell lines. When pLKO.1-mCherry vector was used, the infected cells were sorted by FACS at least twice to purify the stable infectants.

### Quantitative Real-Time PCR (qRT-PCR) and RNA sequencing

Total RNA was extracted by Trizol (Takara, Beijing, China) and reverse-transcribed into cDNA according to the manufacturers' recommendations (Vazyme). Quantitative real-time PCR (qRT-PCR) was performed using a SYBR Green Kit (Vazyme) on an ABI-7500 device (Applied Biosystem). TATA-binding protein (TBP) was used as internal control. qRT-PCR primers are listed in **Table S3**. The extracted total RNA of SUM149-CTRL, SUM149-NICD4, MCF-7-CTRL and MCF-7-NICD4 was sequenced and analyzed at Singeron Biotechnology (Nanjing, China).

### Chromatin Immunoprecipitation (ChIP) Assay

Quantitative ChIP analysis was performed as previously described 20. Cells were cultured to 80-90% confluency and then crosslinked with 1% formaldehyde (Sigma) at RT for 10 min. After neutralization with glycine (125 nmol/L, Sangon), cells were lysed in SDS lysis buffer. The antibody against FLAG (Sigma) was used to pull down the DNA and protein complex, and the purified DNA was subjected to qRT-PCR. We designed primers encompassing the predicted RBP-Jk binding sites in promoter regions of SLUG and GAS1. Subsequently, 1% of total chromatin supernatant before immunoprecipitation was used as input. The primers used were provided in **Table S4**.

### Luciferase reporter assay

The wild-type or corresponding mutated versions of SLUG and GAS1 promoters were cloned into *KpnI & NheI* digested psiCHECK2 vector to replace the original SV40 promoter (Promega, WI, USA). Mutants were generated by overlapping PCR. The primers were listed in **Table S5**. To determine the promoter activity with or without NICD4, HEK293T cells were seeded in 96-well black polystyrene microplate (Corning, NY, USA) and then the promoter reporter plasmids were co-transfected with NICD4 overexpressing plasmid or empty plasmid using lipo3000 (Invitrogen) according to the manufacturer's instructions. 36 hours after transfection, luciferase activity was measured according to the manufacturer's instructions (Promega).

### Mammosphere formation assay

For primary and second mammosphere formation assay, 50,000 of SUM149 and 10,000 MDA-MB-231 cells were plated onto 6-well ultra-low attachment plates (Corning). Cells were cultured in complete MammoCult^TM^ medium kit (STEMCELL, MA, USA) for 10 days. The number of spheres were counted under microscope with a threshold diameter of 100 um and representative pictures were taken. For secondary mammosphere formation assay, the primary spheres were collected and digested by 0.25 % trypsin at 37°C and plated as previously described 20. For serial dilution mammosphere formation assay, cells were plated into 96-well ultra-low attachment plates (Corning) at a series of dilution (10, 100, 1,000 and 10,000) and cultured for 10 days. Half culture medium was exchanged every two days for each well. Sphere number was counted under microscope. The data was analyzed and plotted using the ELDA tool 30 (http://bioinf.wehi.edu.au/software/elda/).

### Immunohistochemistry Staining and Semi-quantitation

Patient breast cancer tissues and their corresponding adjacent normal tissues were obtained from Shanghai cancer hospital affiliated with Fudan University. The sections of paraffin-embedded human tissues were dewaxed and rehydrated in xylene and graded alcohol solutions. Anti-NOTCH4 (1:100, CST) primary antibody was used to stain NOTCH4. The expression of NOTCH4 in breast cancer tissues was assessed in terms of the intensity of immunostaining and scored based on the following four grades: 0=absent; 1=weekly positive; 2=moderate; 3=strong. We counted and graded up to 500 cells of epithelial cells in all samples. The immunohistochemical score (H-score) was calculated using the following equation: H-score=Σ(1+*i*) ×P*_i_*× 100, where *i*=0,1,2,3 and P*_i_* is the proportion of cells exhibiting the relevant staining intensity 31.

### MTT cell proliferation assay

1000 of SUM149 or 500 of MDA-MB-231 cells were seeded into each well of 96-well plates, with 6 replicates for each group and detected at the 3^rd^, 5^th^, and 7^th^ day. At the indicated time points, 20 μL of MTT stock solution (5 mg/mL, Biosharp, China) was added into each well and incubated for 4 hours at 37°C. Subsequently, the supernatant was removed and 100 μL DMSO was added to dissolve the purple product formazan by gently shaking the plates at RT for 10 min. The optical density at 490 nm (OD490) was measured and the values obtained at the 3^rd^, 5^th^, and 7^th^ day were normalized to the 3^rd^ day.

### Chemoresistance assay

MTT assay technique was used to assess the cytotoxicity effect of Docetaxel (DOC) in cell lines used in this study. 5000 SUM149 or 3000 MDA-MB-231 cells per well in 96-well plates were plated and allowed to attach for 24 h. Subsequently, medium was changed with fresh medium containing a serial of concentrations of DOC and cultured for 3 days followed by MTT assay. IC50 value was estimated from dose-response curves obtained using GraphPad Prism 6.0. At least three independent experiments were carried out.

### Transwell assay

In general, 50,000 of SUM149 or 20,000 of MDA-MB-231 cells were carefully dropped into upper wells (Corning) pre-coated with 15% matrigel (BD, NJ, USA) and incubated at 37°C for 36 h with complete medium but devoid of FBS, during which the lower wells were filled with complete medium. Then the cells in the upper wells were carefully wiped off and the upper wells were fixed with methanol-acetic acid mixture (3:1) for 30 min, followed by staining with 0.5% crystal violet for 30 min at RT. Three independent experiments were performed at triplicates. Data was presented as Mean±SD.

### ALDEFLUOR assay and flow cytometry

ALDEFLUOR assay was performed following the manufacturer's protocol (STEMCELL). For staining of fluorescence-conjugated antibodies, namely FITC-CD24 (1:40), PE-CD44(1:20) and APC-NOTCH4 (1:40, BioLegend, CA, USA), against certain cell surface markers, cells suspended in antibody diluent was incubated on ice for 30 min and then washed twice with cold PBS. When combined staining of ALDEFLUOR assay and NOTCH4 was performed, ALDEFLUOR assay was carried out first. For cell cycle analysis, cells were fixed in 70% alcohol at 4 °C for at least 4 h, followed by staining with propidium iodide (Sigma) in the presence of 1% RNase A (Takara) at 37 °C for 30 min. For apoptosis analysis, cells were stained with propidium iodide (Sigma) and APC-Annexin V (BD), according to the manufacturer's recommendations. Analysis of the samples were completed on Moflo Astrios or CytoFlex (Beckman Coulter), equipped with a 405/448 nm channel for DAPI to delineate dead cells, a 488/513 nm channel for ALDEFLUOR or FITC, a 561/579 nm channel for PE, and a 640/671 channel for APC detection.

### Western blotting analysis

Total cell protein was extracted by lysing cell pellets with RIPA lysis buffer supplemented with protease inhibitor cocktail (Beyotime), followed by quantifying with BCA kit (Thermo Fisher, NY, USA). Equal amount of total protein of each sample was separated by 10% SDS-PAGE and transferred to PVDF membrane. Antibodies used were listed in **Table S6.** Signal was detected using Western Chemiluminescence Substrate (Millipore, MA, USA) on chemiluminescence apparatus (SAGE, Beijing, China).

### Statistics

All data were presented as the mean ± SD unless otherwise specified. Date analyses were performed using Student's *t*-test or one-way ANOVA test with GraphPad Prism Version 6.0e (CA, USA). p<0.05 was considered statistically significant and the p-values in figures were indicated with stars as follows: * p<0.05, ** p<0.01, *** p<0.001.

## Results

### NOTCH4 is highly expressed in TNBC patients and predicts poor prognosis

**Figure 1:**
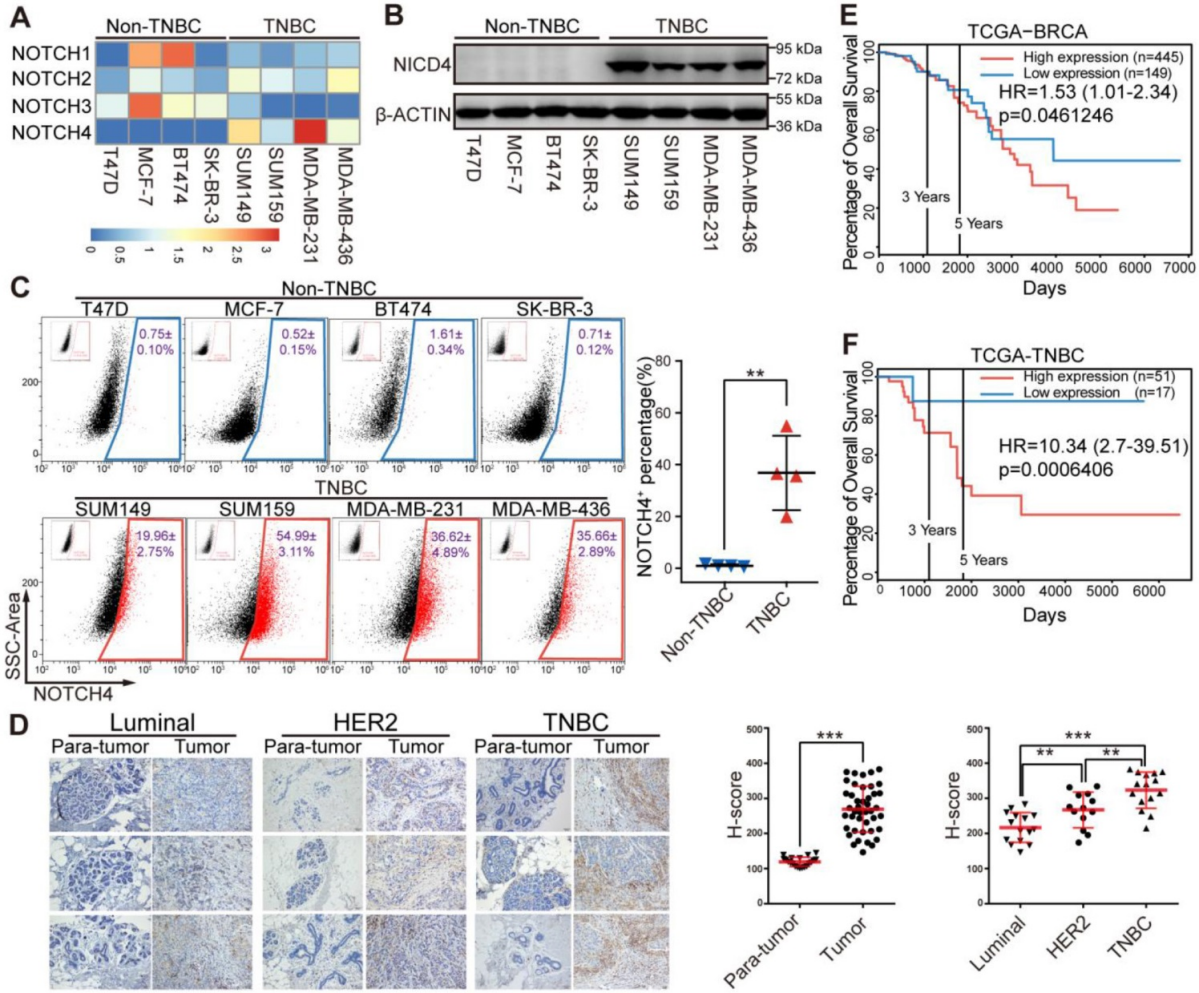
**NOTCH4 was highly expressed in TNBC cell lines and indicated poor prognosis in TNBC patients. (A)** Quantitative real-time PCR was performed to determine the mRNA expression level of NOTCH1-4 in four Non-TNBC cell lines and four TNBC cell lines, with TBP used as internal control. The relative-expression data was normalized to generate the heatmap.** (B)** NICD4 was detected by western blotting, with β-ACTIN as loading control.** (C)** Flow cytometry analysis was performed to determine the level of cell surface NOTCH4. Representative pictures of three independent experiments were shown (left), NOTCH4^+^ percentage was shown as Mean±SD (right). **(D)** IHC staining of paraffin sections in breast tumors and para-tumor tissues from patients with antibody specific to NOTCH4, Scale bar: 50 μm (left). Semi-quantitative IHC score (H-score) was calculated to indicate NOTCH4 expression in breast tumors and para-tumors (right). **(E-F)** Overall survival analysis of NOTCH4 expression in clinical RNA-seq data from TCGA at PROGgene-V2 (http://genomics.jefferson.edu/proggene/),** (E)** unsorted dataset of BRCA and** (F)** TNBC-limited subset, with Hazard Ratio (HR) and p-value indicated. * P<0.05, ** P<0.01, *** P<0.001.

Since the four NOTCH receptors were reported to exert diverse functions in physiological and pathological processes including cancer, we propose that they might play differential roles in breast cancer. We determined the mRNA level of NOTCH receptors in breast cancer cells and observed that NOTCH4 was highly expressed in TNBC cells (**Figure 1A**), implying an important role of NOTCH4 in TNBC. This TNBC-specific expression pattern was further observed at protein level in western blotting (**Figure 1B**). Interestingly, the accumulation of NICD4 implied that NOTCH4 was aberrantly activated in TNBC cells. We next stained cell surface NOTCH4 for flow cytometry analysis and observed that TNBC cells contained a significantly higher proportion of NOTCH4^+^ population compared with non-TNBC cells (**Figure 1C**) (NOTCH4^+^ population accounting for 19.96±2.75% in SUM149, 54.99±3.11% in SUM159, 36.62±4.89% in MDA-MB-231 and 35.66±2.89% in MDA-MB-436). Our discovery of the enriched NOTCH4^+^ population was in consistence with the report of Rosemarie et al, who used a NOTCH4 activity reporter and found that NOTCH4 activity was higher in TNBC cell lines 32.

We then performed immunohistochemistry staining of NOTCH4 in patients' breast tumors and corresponding para-tumor tissues and revealed that NOTCH4 was highly expressed in tumors compared with para-tumor tissues, demonstrating the dysregulated expression of NOTCH4 in breast cancer. Again, we observed significant upregulation of NOTCH4 in TNBC, in comparison with other two subtypes (Luminal and HER2^+^) of breast cancer (**Figure 1D**). The higher expression of NOTCH4 in TNBC agreed with previous reports 26, 27. Our further analysis of public database indicated that higher expression level of NOTCH4 significantly correlated with poorer overall survival of breast cancer patients (**Figure 1E, Figure S1A-S1B**). This negative correlation was even more remarkable when we focused on the TNBC cohort (**Figure 1F**). These data together suggested that aberrant expression and activation of NOTCH4 might promote TNBC malignancy.

### NOTCH4 enriches BCSCs in TNBC

**Figure 2:**
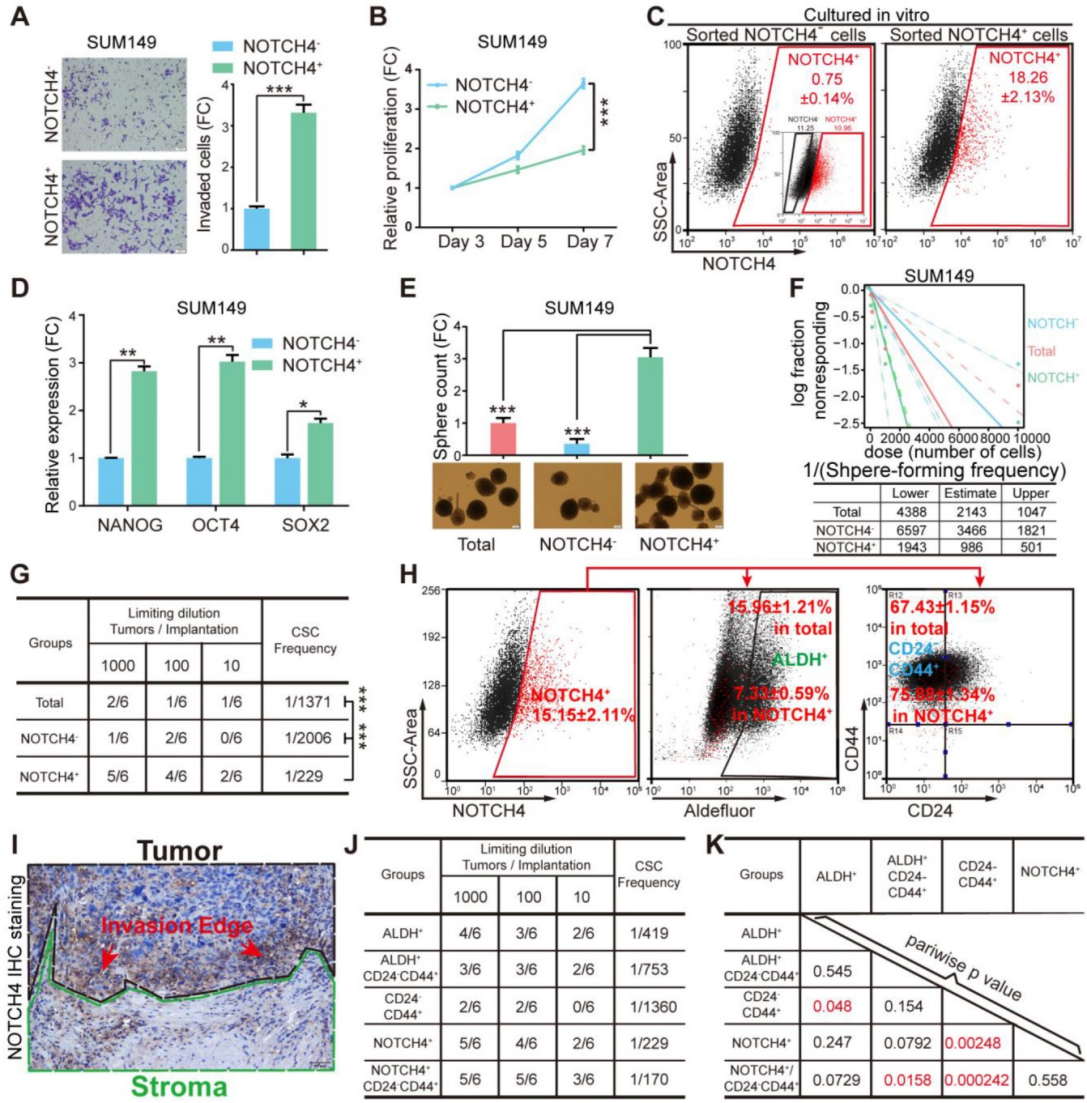
**NOTCH4 enriched BCSCs in TNBC cells**. **(A-F)** NOTCH4^+^ and NOTCH4^-^ cells were sorted from SUM149 by FACS to measure the following characteristics: **(A)** invasion ability by transwell assay; **(B)** proliferation ability by MTT assay;** (C)** *in vitro* differentiation ability by flow cytometry with the initial FACS graph showed as an insert; **(D)** fold change of stemness gene expression level by qRT-PCR, with TBP used as internal control;** (E)** mammosphere formation ability, Scale bar: 100 μm. **(F)** Serial dilution assay was performed to determine the sphere-formation frequency; data was calculated by the online tool ELDA. **(G)** Sorted SUM149 cells were transplanted in situ into nude mice at indicated serial dilutions to assess the BCSC frequency; data was calculated by the online tool ELDA (http://bioinf.wehi.edu.au/software/elda/). **(H)** NOTCH4, ALDEFLUOR and CD24/CD44 were co-stained to determine the percentage of overlapping population in SUM149. **(I)** A representative picture of IHC staining of NOTCH4 in TNBC paraffin sections, using dashed lines to indicate the tumor and stroma edges. **(J-K)** Comparison of BCSC enrichment ability between NOTCH4 and the present commonly used BCSC markers as well as their combinations, using serial transplantation assay of SUM149, CSC frequency **(J)** and pairwise p values **(K)** were calculated. Triple independent experiments were carried out and representative results were shown. * P<0.05, ** P<0.01, *** P<0.001.

Given previous reports indicating NOTCH4 to be involved in BCSC regulation 24, 33, we asked whether the NOTCH4^+^ cells would show characteristics of BCSCs. To explore this, we first sorted the NOTCH4^-^ and NOTCH4^+^ subpopulations by FACS to determine their invasiveness, proliferation and tumorigenesis ability. We found that the NOTCH4^+^ population were more invasive but less proliferative than the NOTCH4^-^ counterpart in both SUM149 and MDA-MB-231 (**Figure 2A-2B, Figure S2A-S2B**). Furthermore, NOTCH4^+^ cells in both cell lines were more potent in tumorigenesis *in vivo* (**Figure S2C**). Besides, NOTCH4^+^ MDA-MB-231 cells showed stronger lung metastasis ability (**Figure S2D**) in contrast to NOTCH4^-^ cells. These results suggested that the NOTCH4^+^ cells were endowed with more malignant features.

To determine whether the NOTCH4^+^ population enriched BCSCs, we sorted NOTCH4^+^ and NOTCH4^-^ cells and cultured them *in vitro* for 10 days, followed by FACS analysis. We observed that the sorted NOTCH4^+^ cells recapitulated the whole population including both NOTCH4^+^ cells and NOTCH4^-^ cells, with a similar ratio to that of the initial SUM149 cell culture (**Figure 1C, 2C**), whereas NOTCH4^-^ cells rarely generated NOTCH4^+^ cells. These results suggested that the NOTCH4^+^ population enriched CSCs that were self-renewal to maintain the CSC pool, as well as to differentiate to generate more differentiated progenies. We then detected the expression of stemness factors (NANOG, OCT4, SOX2) in NOTCH4^-^ and NOTCH4^+^ populations. As expected, we observed higher levels of stemness factors in NOTCH4^+^ cells (**Figure 2D, Figure S2E**), implying stronger stemness in NOTCH4^+^ population. Furthermore, the NOTCH4^+^ cells of both SUM149 and MDA-MB-231 exhibited higher sphere-formation frequency in serial dilution mammosphere assay, demonstrating that the NOTCH4^+^ population enriched CSCs *in vitro* (**Figure 2E-2F, Figure S2F-S2G**). To further demonstrate that the NOTCH4^+^ subpopulation enriched CSCs *in vivo*, we performed serial dilution tumor transplantation using SUM149 to assess the CSC frequency of the sorted NOTCH4^-^, NOTCH4^+^, and total populations. The significantly higher CSC frequency of the NOTCH4^+^ population reflected enriched CSCs (**Figure 2G, Figure S2H**). Taken together, these results demonstrated that NOTCH4 enriched BCSCs that were characterized to be potent in tumorigenesis and invasion, but slow in proliferation. Accordingly, NOTCH4^+^ could be used as a BCSC marker in TNBC.

### NOTCH4 is an efficient ML-BCSC marker in TNBC

Since the NOTCH4 enriched BCSCs were shown to be more invasive and less proliferative, regarding to our previous work declaring the two states of CSCs 14, the NOTCH4^+^ cell population was likely to adapt a mesenchymal-like status with enhanced invasiveness and quiescent proliferation. Whereupon, we wondered the similarity or difference between the NOTCH4^+^ marked BCSC population and the previously reported BCSCs enriched by CD24^-^CD44^+^ or ALDH^+^. To illuminate this, we co-stained NOTCH4 with CD24/CD44 or ALDH for flow cytometry analysis. Interestingly, NOTCH4^+^ population was observed to overlap more with CD24^-^CD44^+^ population, but less with the ALDH^+^ population (**Figure 2H, Figure S2I**). Furthermore, when we re-examined our IHC staining of NOTCH4, the NOTCH4^+^ cells were more frequently found to localize near the interface of tumor and stroma (**Figure 1D, 2I**), indicating a pro-invasion role of NOTCH4. These data support that NOTCH4^+^ cells are in a mesenchymal-like state and thus might promote invasion and metastasis.

We have previously shown that TNBC cells are almost 100% CD44^+^ 15, therefore NOTCH4^+^ might be a more promising CSC marker alternative to CD24^-^ CD44^+^ in TNBC. To explore this possibility, we performed tumor transplantation in a serial dilution to compare the tumor formation frequency of the following BCSC enriched populations, namely ALDH^+^, ALDH^+^CD24^-^CD44^+^, CD24^-^CD44^+^, NOTCH4^+^ and CD24^-^CD44^+^NOTCH4^+^. Our results showed that the tumorigenesis frequency of NOTCH4^+^ population was slightly higher than that of ALDH^+^ population. Nevertheless, NOTCH4^+^ population displayed significantly more efficient tumorigenesis than CD24^-^CD44^+^ population. More excitingly, the addition of CD24^-^CD44^+^ further improved the tumorigenesis efficiency of NOTCH4^+^ population, although with only a slight degree (**Figure 2J-2K, Figure S2J**). Another interesting characteristic of CD24^-^CD44^+^ ML-BSCSs is their chemoresistance, which has been reported to be conferred by both cell cycle quiescence 34, 35 and EMT 36, 37, and these were observed in NOTCH4^+^ cells in our results. To determine whether NOTCH4^+^ cells were endowed with enhanced chemoresistance, we tested the sensitivity of sorted cells to docetaxel (DOC). We found that NOTCH4^+^ cells were relatively more resistant to docetaxel than the NOTCH4^-^ population, as indicated by increased IC50 concentration (**Figure S2K**). These results demonstrated that NOTCH4 enriched BCSCs with similar traits of the previously defined ML-BCSCs characterized by CD24^-^CD44^+^ in TNBC, but with a remarkably higher efficiency.

### Overexpression of NICD4 mimicked the phenotypes of NOTCH4^+^ cells in TNBC cell lines

**Figure 3:**
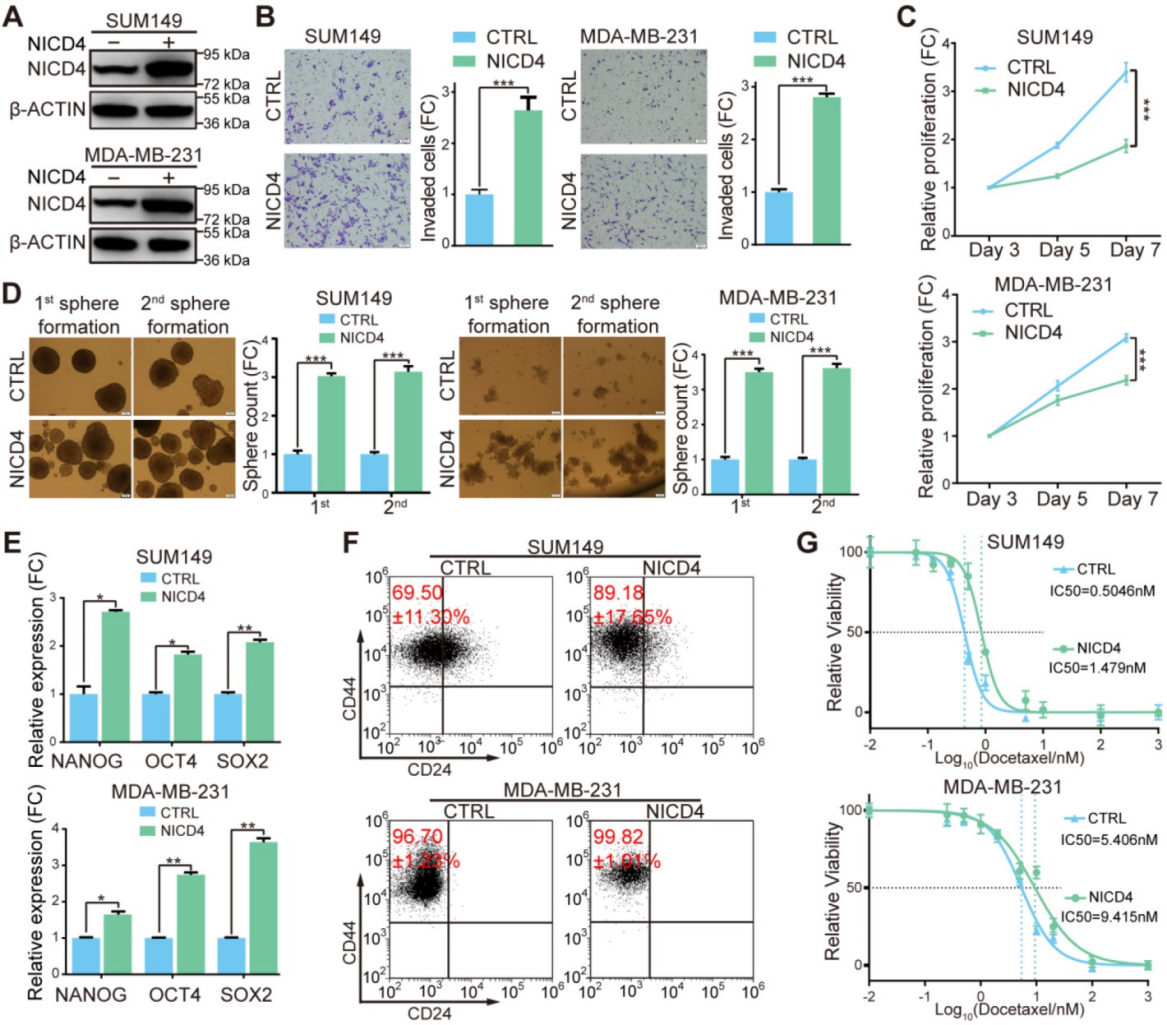
** Overexpression of NICD4 induced ML-BSCS characteristics. (A)** NICD4 overexpression was confirmed in TNBC cell lines SUM149 and MDA-MB-231 by western blotting, with β-ACTIN used as loading control.** (B-G)** The biological functions of NICD4 overexpression in SUM149 and MDA-MB-231 cells were assessed: **(B)** invasion ability by transwell assay, **(C)** proliferation by MTT assay, **(D)** primary and secondary mammosphere formation ability, **(E)** master stemness regulating transcription factors expression level, **(F)** CD24^-^CD44^+^ BCSC percentage, and **(G)** chemosensitivity to docetaxel by MTT assay. Triple independent experiments were carried out and representative results were shown. * P<0.05, ** P<0.01, *** P<0.001.

To further explore the underlying mechanisms of NOTCH4 in regulating BCSCs, we constructed NOTCH4 intracellular domain (NICD4)-overexpressing TNBC cell lines in SUM149 and MDA-MB-231 (**Figure 3A**). Functionally, we observed significantly enhanced invasion in NICD4-overexpressing cells (**Figure 3B**). However, upon NICD4 overexpression both cell lines suffered from dramatically inhibited proliferation (**Figure 3C**), which did not result from elevated apoptosis (**Figure S3A**), but might resulted from cell cycle arrest at G0/G1 phase (**Figure S3B**), similar to previous observations in other cancer cells 38, 39. Besides, NICD4 overexpression increased mammosphere formation ability in both primary and secondary mammosphere formation assay (**Figure 3D**). Furthermore, NICD4 upregulated the stemness-associated transcription factors in SUM149 and MDA-MB-231 (**Figure 3E**). NICD4 also increased CD24^-^CD44^+^ proportion in SUM149, whereas not in MDA-MB-231 that was shown to be almost 100% CD24^-^CD44^+^ (**Figure 3F**) 15. Moreover, docetaxel chemoresistance ability of the NICD4-overexpressing cells was found to be reinforced (**Figure 3G**). Interestingly, when we determined NICD4 expression in two taxane-resistant SUM159 cell lines established in our lab, remarkably elevated NICD4 level was observed (**Figure S3C**). These results indicated that NOTCH4 activation was involved in chemoresistance of TNBC.

On the contrary of overexpression, we tried to inhibit NOTCH4 activity to determine whether the above-mentioned effects could be reversed. We abandoned the usage of γ-secretase inhibitors (GSIs) because NOTCH4 activation was reported to be merely inhibited by GSIs 20, 24. Therefore, we used shRNA mediated knockdown. However, probably due to the relatively low endogenous NOTCH4 mRNA level in breast cancer, we could only achieve a partial knockdown efficiency (**Figure S3D**). Nevertheless, we observed that even partial knockdown of NOTCH4 resulted in observable proliferation inhibition (**Figure S3E**). To understand why knockdown of NOTCH4 displayed a similar proliferation-inhibiting phenotype to NICD4 overexpression, we analyzed apoptosis and cell cycle and found that the reduced cell proliferation was not due to cell cycle arrest but as a result of elevated apoptosis (**Figure S3F**). Furthermore, the incomplete interference of NOTCH4 showed a moderate inhibitory effect on mammosphere formation ability and invasion ability in both SUM149 and MDA-MB-231 cells, but significantly impaired chemoresistance (**Figure S3G-S3I**). These results implied that NOTCH4 pathway might play an important role in maintaining BCSC survival through inhibiting apoptosis.

### NICD4 overexpression induced an expression profile associated with EMT and cellular quiescence in TNBC cells

**Figure 4:**
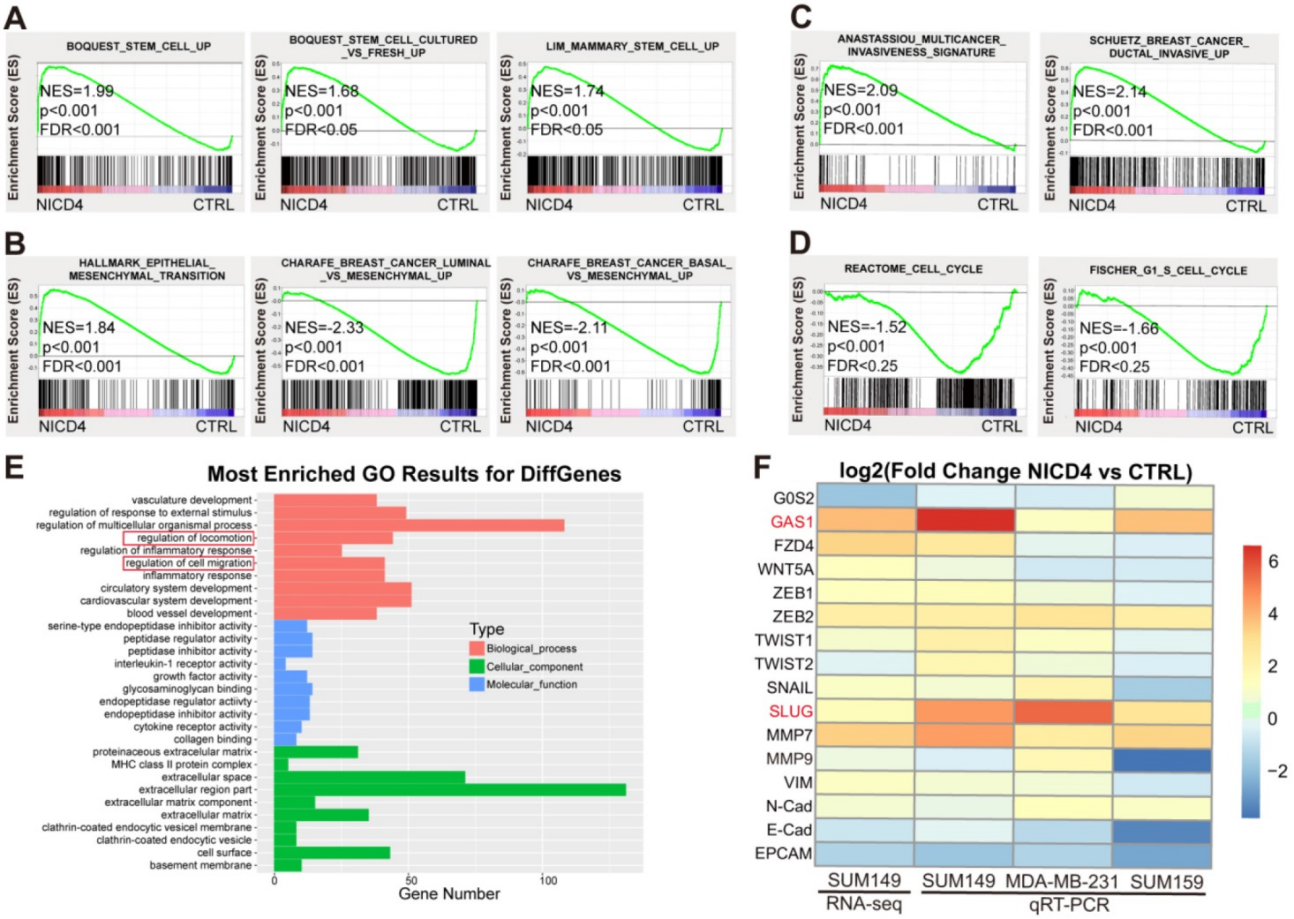
** Activated NOTCH4 (NICD4) induced an ML-BCSC expression profile. (A-D)** GSEA analysis of RNA-seq data of NICD4 overexpressing SUM149 exhibited enrichment of **(A)** stemness regulating genes, **(B)** mesenchymal-like transition associated genes, **(C)** invasion enhancing genes and **(D)** cell cycle arrest associated genes. **(E)** GO analysis of the RNA-seq data indicated enriched motility associated genes. **(F)** qRT-PCR was performed to validate the significantly changed genes associated with cell cycle arrest and EMT related genes in 3 TNBC cell lines overexpressing NICD4, including SUM149, MDA-MB-231 and SUM159. The RNA-seq results of SUM149 were also presented in the heatmap for comparison. TBP was used as internal control. Data was shown as log_2_ of fold change relative to the control group of each cell line.

To explore the underlying mechanisms of NOTCH4 in regulating TNBC ML-BCSCs, we performed RNA-seq of NICD4-overexpressing SUM149. In accordance with the above phenotypes, we observed that the profile of NICD4-overexpressing group enriched for genes associated with enhanced “stemness” (**Figure 4A**) using GSEA analysis 40, indicating that NOTCH4 promoted self-renewal of BCSCs. Besides, we also identified an EMT profile (**Figure 4B**), an enhanced invasion profile (**Figure 4C**) and a cell cycle arrest signature (**Figure 4D**). Furthermore, Gene Ontology (GO) analysis also enriched for pathways related to mobility, corresponding to the elevated invasion capacity of NICD4-overexpressing cells (**Figure 4E**). These results confirmed our previous functional assays at transcriptomic level. Next, we performed qRT-PCR to confirm the most significantly changed genes associated with EMT and cell cycle regulation in the RNA-seq results in three TNBC cell lines, namely SUM149, MDA-MB-231 and SUM159. We found that SLUG and GAS1 were consistently and most dramatically upregulated in NICD4-overexpressing cells (**Figure 4F**), suggesting that NOTCH4 might promote cell invasion via SLUG and induce quiescence through GAS1.

However, analysis of the RNA-seq data of a non-TNBC cell line, MCF-7, did not display the above-described mesenchymal-like stem features. Surprisingly, NICD4 overexpression seemed to comprehensively suppress the expression of stemness associated genes, promote neuron-like differentiation, and inhibit invasiveness and mesenchymal-like features in MCF-7 (**Figure S4A-S4E**). GO analysis also revealed similar portraits, as NICD4 overexpression in MCF-7 resulted in significant enrichment of negative regulators of cell mobility and genes toward neuron-like differentiation (**Figure S4F**). Therefore, NICD4 seemed to exert quite different effects on the molecular and phenotypic portraits in TNBC and non-TNBC cells, suggesting the function of NOTCH4 was cell-context dependent.

Taken together, activated NOTCH4 induced mesenchymal-like features in TNBC cells, consistent with its reported roles in promoting TNBC invasion and metastasis, which might be mediated by SLUG and GAS1.

### NICD4 transcriptionally upregulates SLUG to induce EMT

**Figure 5:**
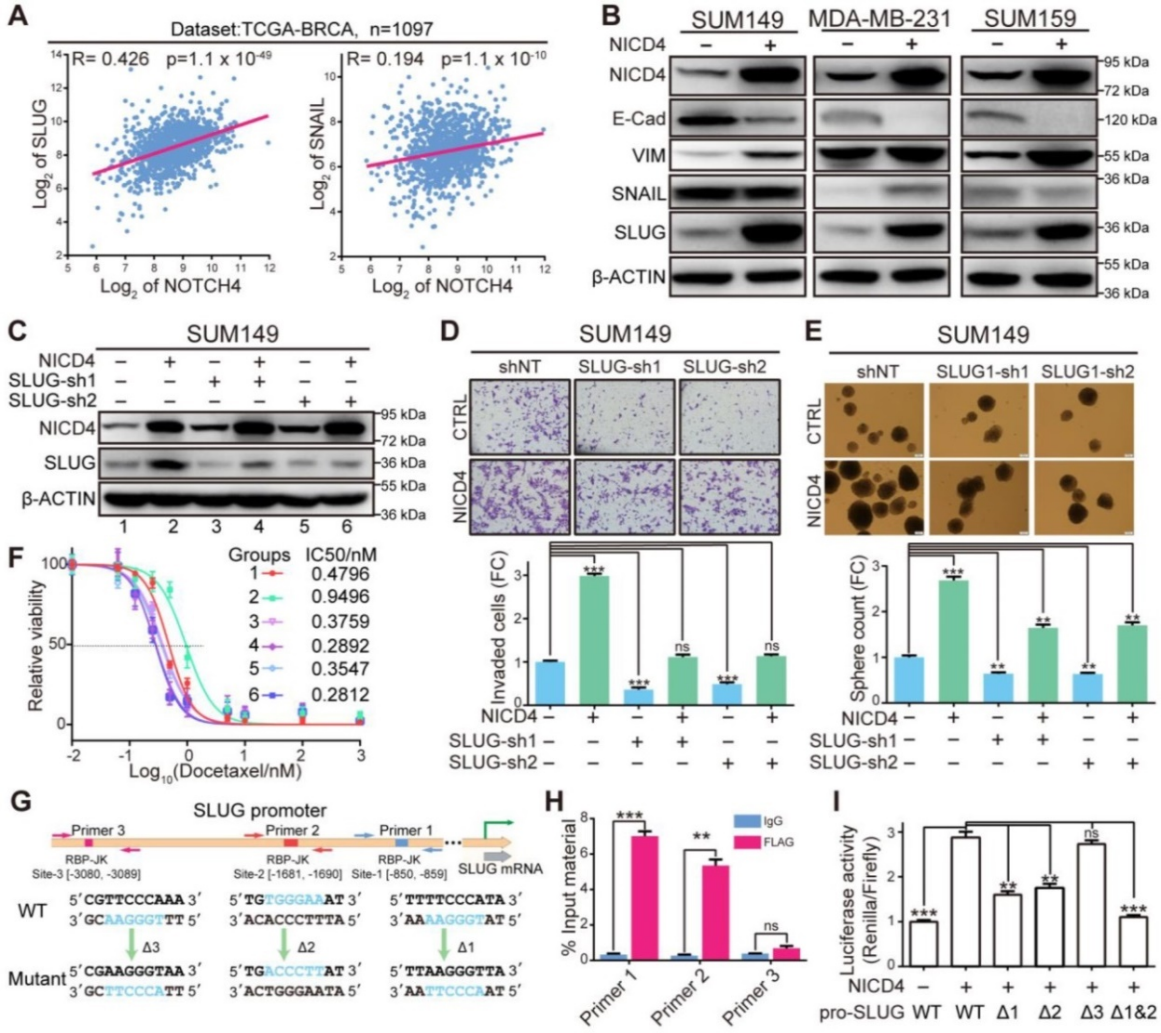
** NICD4 directly upregulated SLUG expression to induce EMT. (A)** Gene expression correlation between NOTCH4 and SLUG or SNAIL was plotted via R2 (https://hgserver1.amc.nl/cgi-bin/r2/main.cgi) using dataset from TCGA-BRCA. **(B)** Markers for EMT (VIM, SNAIL and SLUG) and MET (E-Cad) were detected at protein level in NICD4-overexpressing SUM149, MDA-MB-231, and SUM159. **(C)** SLUG was interfered by shRNA in NICD4 overexpressing cells or control cells. The knockdown efficiency was determined by western blotting, using β-ACTIN as loading control. **(D-F)** We then determined** (D)** invasion ability by transwell assay, **(E)** mammosphere formation capability and **(F)** chemosensitivity via MTT assay after SLUG knockdown in SUM149 cells, the groups represented by numbers in** (F)** were the same as in **(C)**.** (G)** Schematic depiction of the SLUG promoter with putative binding sites (colored rectangles) indicated. Primer sets for ChIP analyses were indicated by the arrows and the designed mutants were shown below.** (H)** ChIP and qRT-PCR were subsequently performed. The results of each primer were expressed as % Input material. IgG was used as control. **(I)** Dual-luciferase reporter assays of the SLUG promoter region. The relative luciferase signal ratio (Relina Luc. /Firefly Luc.) was further normalized to that of the group transfected with control vector. Triple independent experiments were carried out and representative results were shown. ns=not significant, ** P<0.01, *** P<0.001.

SLUG has long been reported as a master transcription factor in regulating EMT both physiologically and pathologically 41, and demonstrated to be a direct target of NOTCH4 in cardiac development where it plays important roles in regulating EMT during cardiac cushion cellularization 42. Therefore, we hypothesized that NOTCH4 might induce EMT and invasion via upregulating SLUG in TNBC. To demonstrate this, we firstly analyzed the correlation between NOTCH4 and SLUG in breast cancer database online (http://r2.amc.nl). We found that NOTCH4 showed a significantly positive correlation with SLUG but not SNAIL (the SLUG congener) (**Figure 5A, Figure S5A-S5B**), implying a specific regulation of NOTCH4 on SLUG but not SNAIL. We then performed western blotting to detect a panel of EMT and MET markers, including SNAIL and SLUG (**Figure 5B**). We still observed dramatic upregulation of SLUG but not SNAIL at protein level, indicating the specific regulation role of NICD4 on SLUG. Besides, we observed expected changes of other EMT-MET markers, such as downregulated E-Cadherin (E-Cad) and upregulated VIMENTIN (VIM), suggesting a transition towards mesenchymal-like state. Interestingly, knockdown of SLUG in NICD4-overexpressing cells significantly reversed the NICD4 enhanced invasion, and incredibly impaired the mammosphere formation and chemoresistance ability (**Figure 5C-5F, Figure S5C-S5F**). Unexpectedly, we observed significantly elevated apoptosis in NICD4 overexpressing cells after SLUG knockdown (**Figure S5G**), implying that SLUG was involved in the anti-apoptotic role of NOTCH4 (**Figure S3F**). Besides, SLUG overexpression was shown to induce enhanced invasion and stemness significantly in TNBC cells (**Figure S5H-S5J**).

To determine how NOTCH4 regulated SLUG expression in TNBC, we investigated the SLUG promoter region for potential RBP-Jk binding sites using JASPAR 43 and obtained three potential sites (**Figure 5G**). To identify the genuine binding sites, we firstly performed ChIP assay against FLAG-tagged NICD4 and found that site-1 and site-2 were significantly enriched (**Figure 5H**). Furthermore, we cloned the promoter region of SLUG into a dual-luciferase reporter vector and mutated the potential binding sites one by one or simultaneously, which were then co-transfected with NICD4-overexpressing plasmid into HEK293T cells. We observed that the mutations in either site-1 or site-2 could partially attenuate the NICD4-upregulated luciferase activity and simultaneous mutation of both sites almost completely abolished the induction of SLUG promoter activity by NICD4 (**Figure 5I**). These results demonstrated that SLUG was a direct target of NOTCH4. Taken together, we concluded that NOTCH4 induced EMT via direct transcriptional activation of SLUG in TNBC. Our data also suggested an important role of SLUG in protecting NOTCH4^+^ cells from apoptosis.

### NICD4 induces cellular quiescence via transcriptionally upregulating GAS1

**Figure 6:**
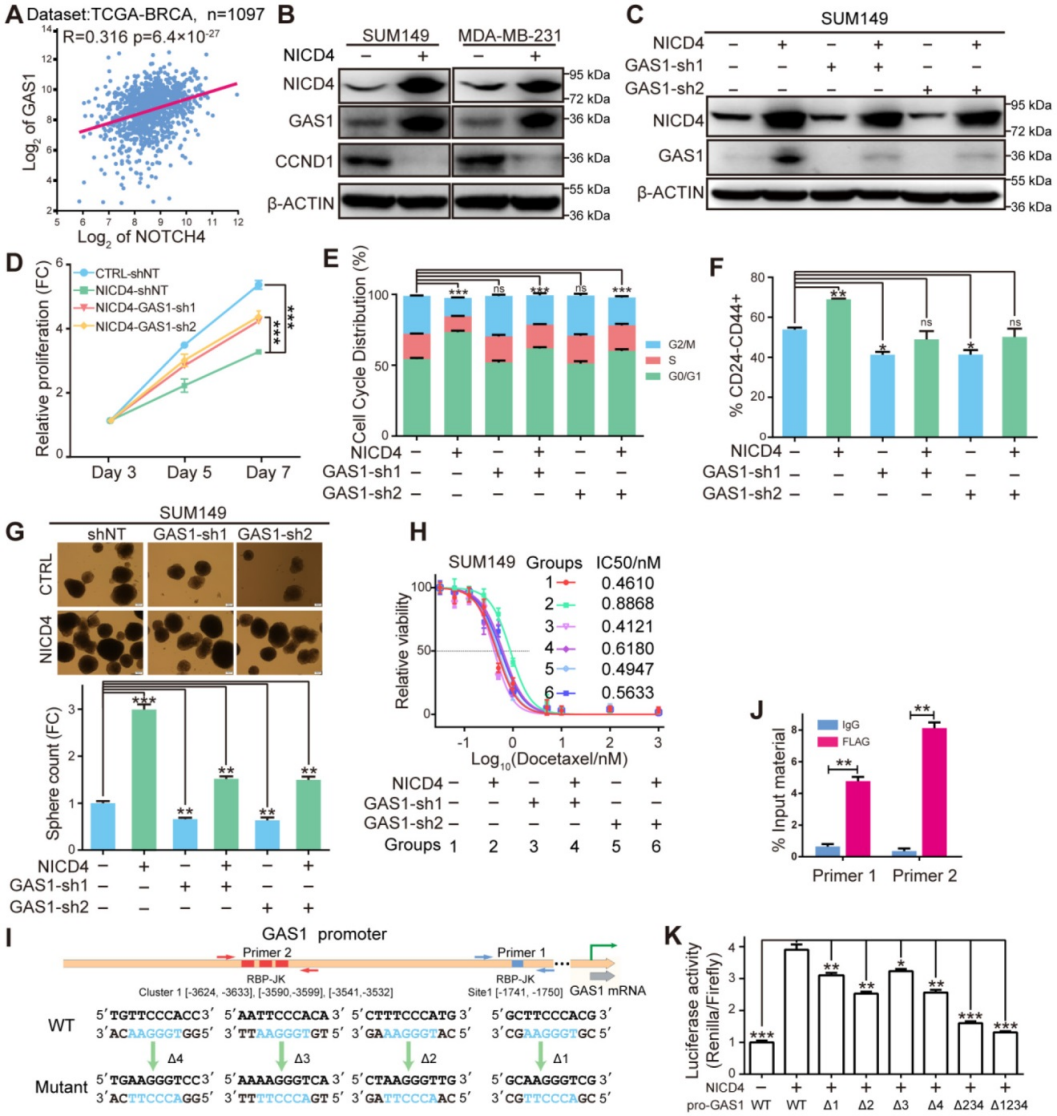
** NICD4 induced cell cycle quiescence via transcriptionally activating GAS1. (A)** Expression correlation between NOTCH4 and GAS1 was plotted using above the mentioned tool R2.** (B)** Induction of GAS1 protein by NICD4 overexpression and expression of Cyclin D1 (CCND1) was determined by western blotting, using β-ACTIN as loading control. **(C)** GAS1 was interfered by lentiviral shRNA. The knockdown efficiency was determined by western blotting, using β-ACTIN as loading control. **(D-H)** In SUM149, we then determined **(D)** cell proliferation by MTT assay, **(E)** cell cycle distribution by flow cytometry, **(F)** CD24^-^CD44^+^ percentage by flow cytometry, **(G)** mammosphere formation capability and **(H)** chemosensitivity by MTT assay, after GAS1 knockdown by shRNA.** (I)** Schematic depiction of the GAS1 promoter to indicate the putative binding sites (colored rectangles). Primer sets for ChIP analyses are indicated by the arrow pairs, with the designed mutants shown below.** (J)** ChIP and qRT-PCR were subsequently performed and the results of each primer was expressed as % Input material. IgG was used as control.** (K)** Dual-luciferase reporter assays of the GAS1 promoter region. GAS1-WT: vector containing the wild-type sequence of the GAS1 promoter. GAS1-Δ234, simultaneous mutation of cluster-1; GAS1-Δ1234, simultaneous mutation of all four predictive RBP-Jk binding sites. The relative luciferase signal ratio (Relina Luc. /Firefly Luc.) was further normalized to that of the group transfected with control vector. ns=not significant, * P<0.05, ** P<0.01, *** P<0.001.

In search for genes responsible for the NICD4 induced cell cycle arrest, we noticed that GAS1 (Growth Arrest-specific Protein 1) was dramatically and consistently upregulated in NICD4-overexpressing cells (**Figure 4F**). GAS1 has been well known for its role in establishing cell cycle arrest at G0/G1 phase by inhibiting DNA synthesis 28, 44. It was later demonstrated to act as a co-receptor of Hedgehog pathway to augment the spreading range of the Shh 29. To explore whether NOTCH4 maintains ML-BCSC dormancy via GAS1, we analyzed the expression correlation of NOTCH4 and GAS1 (http://r2.amc.nl) and found a significant positive correlation (**Figure 6A**, **Figure S6A-S6B**). Moreover, NICD4 overexpression in both SUM149 and MDA-MB-231 resulted in significant induction of GAS1 protein (**Figure 6B**). We also detected the expression of Cyclin D1 (CCND1), whose downregulation has been also regarded as an indicator of G0/G1 cell cycle arrest, and observed significantly reduced CCND1 expression upon NICD4 overexpression (**Figure 6B**). Furthermore, when we knocked down GAS1 in NICD4-overexpressing cell lines (**Figure 6C, Figure S6C**), the inhibited proliferation and cell cycle arrest were rescued (**Figure 6D-6E, Figure S6D-S6E**). Concomitantly, knockdown of GAS1 in NICD4-overexpressing cells also resulted in reduced stemness of TNBC cells, represented by reduced percentage of CD24^-^CD44^+^ population in SUM149 (**Figure 6F**) and mammosphere formation ability in both tested cell lines (**Figure 6G, Figure S6F**). Besides, knockdown of GAS1 partially abolished the NICD4 induced chemoresistance (**Figure 6H, Figure S6G**). Since GAS1 has been reported to enhance Hedgehog pathway, we analyzed our RNA-seq data using GSEA and found an enriched profile of Hedgehog signaling activation in NICD4-overexpressing group (**Figure S6H**), implying that GAS1 might also be involved in NOTCH4^+^ ML-BCSC maintenance through potentiating Hedgehog pathway 45, 46. The role of GAS1 on G0/G1 arrest in TNBC was further confirmed by GAS1 overexpression, which resulted in obviously reduced CCND1 expression and G0/G1 arrest (**Figure S6I-S6J**). However, overexpression of GAS1 alone also resulted in increased apoptosis (**Figure S6K**), which was consistent with previous studies showing that GAS1 was able to induce apoptosis 47, 48.

To determine the regulation manner of NOTCH4 on GAS1 expression, we searched for potential binding sites of RBP-Jk on the promoter region of GAS1 using JASPAR 43 and obtained 4 potential sites (**Figure 6I**). Interestingly, three out of the four sites clustered within a short region of about 100 nucleotides (Cluster-1), implying that GAS1 was very likely to be regulated by RBP-Jk dependent transcription factors. To determine whether GAS1 was directly regulated by NOTCH4, we performed both ChIP and promoter reporter assay as described above. We indeed observed significant enrichment of the predicted regions of both the separate site-1 and the cluster-1, indicating the recruitment of NICD4 to these regions (**Figure 6J**). Subsequent promoter reporter assay demonstrated that all four sites were functional for NICD4 to activate GAS1 expression (**Figure 6K**). In summary, the above results demonstrated that GAS1 was a direct target of NOTCH4 and was essential for NICD4 induced proliferation quiescence.

### SLUG and GAS1 cooperate to maintain the NOTCH4^+^ ML-BCSCs

**Figure 7:**
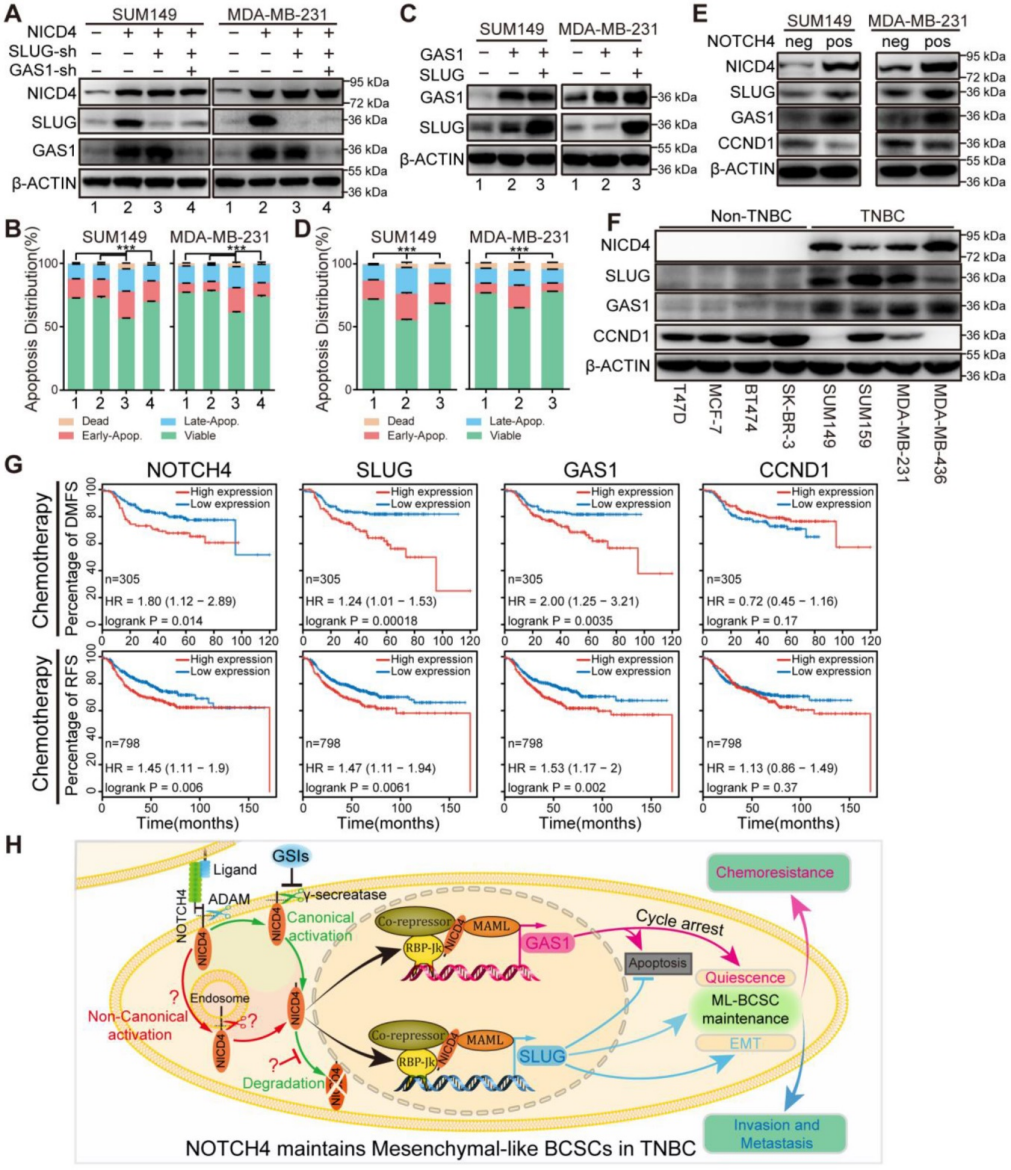
** SLUG and GAS1 cooperated to facilitate NOTCH4 function in maintaining the ML-BCSC characteristics and predicted metastasis and relapse in TNBC patients after chemotherapy. (A)** SLUG alone or both SLUG and GAS1 were knocked down in NICD4-overexpressing SUM149 and MDA-MB-231.** (B)** Apoptosis of the indicated cells in **(A)** was assessed using flow cytometry. **(C)** GAS1 alone or both SLUG and GAS1 were overexpressed simultaneously in SUM149 and MDA-MB-231. **(D)** Apoptosis assay was performed to the indicated cells in **(C)**. Bars showed the percentage of cells distributed in different status, namely, viable cells, early apoptosis (Early-Apop. in short), late apoptosis (Late-Apop. in short) and dead cells. **(E)** Western blotting to determine the expression of the indicated proteins in sorted NOTCH4^neg^ and NOTCH4^pos^ cells of both SUM149 and MDA-MB-231 (neg, negative; pos, positive). **(F)** Western blotting to determine the expression pattern of the indicated proteins in TNBC and non-TNBC cell lines. β-ACTIN was used as loading control. **(G)** DMFS and RFS analysis of NOTCH4, SLUG, GAS1 and CCND1 using Kaplan-Meier tool with cohort received chemotherapy. **(H)** A schematic summary of the findings of this work. NOTCH4 aberrant activation induces and maintains ML-BCSCs status by transcriptionally activating SLUG and GAS1, which work together to endow NOTCH4^+^ cells with enhanced EMT and cellular quiescence, and eventually leading to TNBC metastasis and chemoresistance. *** P<0.001.

In the above results, we have noticed that either knockdown of SLUG in NICD4-overexpressing cells or overexpression of GAS1 by itself induced apoptosis (**Figure S5G, S6K**). These observations suggested a potential of GAS1 in inducing apoptosis, which might be suppressed by SLUG. Indeed, GAS1 has been reported to induce apoptosis in cancer, such as glioma 47 and breast 48, and SLUG has been demonstrated to inhibit apoptosis in breast cancer and is essential for the survival of cancer cells during metastasis 49. As expected, after knockdown of GAS1 following SLUG interference in NICD4-overexpressing cells, the apoptosis induced by SLUG interference was significantly alleviated (**Figure 7A**-**7B**), accompanied by a release of cell proliferation suppression (**Figure S7A**) but a significant decrease in sphere formation ability (**Figure S7B**). Furthermore, introduction of SLUG into GAS1-overexpressing cells obviously attenuated GAS1 induced apoptosis (**Figure 7C-7D**). Given our above results, it was not surprising to observe that co-overexpression of GAS1 and SLUG in parental cells mimicked the effects of NICD4 overexpression, including enhanced invasion, inhibited proliferation, elevated mammosphere formation ability and chemoresistance (**Figure S7C-S7F**).

To further confirm our above results that NOTCH4 upregulated SLUG and GAS1 expression in TNBC cells, we sorted NOTCH4^-^ and NOTCH4^+^ subpopulations to detect the endogenous SLUG and GAS1 expression. Expectedly, we observed higher expression of both SLUG and GAS1 in NOTCH4^+^ cells compared with the NOTCH4^-^ counterpart (**Figure 7E**). We also confirmed the expression of NOTCH4, SLUG, GAS1 and CCND1 in different subtypes of breast cancer cell lines and observed expected expression patterns. NOTCH4 and SLUG were almost exclusively expressed in TNBC cell lines and GAS1 was expressed at higher level in TNBC cell lines, whereas CCND1 expression was lower in TNBC cell lines (**Figure 7F**)**.** We expanded this observation to more cell lines by analyzing the RNA-seq data retrieved from Cancer Cell Line Encyclopedia (CCLE) (**Figure S7G**), demonstrating that SLUG and GAS1 were positively correlated with NOTCH4 in TNBC cell lines.

We then asked whether our discovery could predict clinical outcome. To answer this question, we utilized the Kaplan-Meier plotter 50 to analyze the association between the expression level and distant metastasis-free survival (DMFS) or relapse free survival (RFS) in breast cancer. We found that each of NOTCH4, SLUG and GAS1 predicted poor DMFS (**Figure S8A**). More interestingly, when focusing on the patients who received chemotherapy, we observed that high expression of each of NOTCH4, SLUG and GAS1 was associated with significantly worse DMFS and RFS (**Figure 7G**), whereas CCND1, downregulated in ML-BCSCs, didn't display such a trend (**Figure 7G, Figure S8A**). Similar results were observed in TNBC patients, although with a weaker significance due to the small cohort (**Figure S8B-S8C**). The potential to predict earlier metastasis and relapse after chemotherapy suggested that the NOTCH4-SLUG-GAS1 circuit was involved in promoting metastasis and chemoresistance.

As illustrated in **Figure 7H**, NOTCH4 was aberrantly highly expressed and activated in TNBC, and could be used to enrich ML-BCSCs. Mechanistically, SLUG and GAS1 were among the direct downstream key effectors, if not only, of NOTCH4 in maintaining the ML-BCSC characteristics. SLUG and GAS1 cooperated to exert the effects of NICD4 in TNBC, including enhanced invasiveness and chemoresistance, as well as cellular quiescence. Moreover, SLUG also functioned to diminish the pro-apoptosis role of GAS1, thus harnessing the previously regarded tumor suppressor gene to serve as a tumor promoting gene.

## Discussion

In this work, we showed that NOTCH4 was significantly upregulated and aberrantly activated in TNBC cells, and that NOTCH4 overexpression was related to reduced overall survival. Hereafter, we demonstrated that NOTCH4^+^ could efficiently enrich BCSCs in TNBC cells, which showed characteristics of ML-BCSCs, such as enhanced invasion and migration, cellular quiescence and elevated chemoresistance. As expected, NOTCH4^+^ BCSCs were shown to overlap with the CD24^-^CD44^+^ subpopulation but not the ALDH^+^ counterpart, and resided at the invasive frontier of breast tumors, consistent with our previous two-state BCSC theory 14. We then performed RNA-seq of NICD4-overexpressing cells and found that constitutive activation of NOTCH4 dramatically triggered a molecular profile of enhanced stemness, EMT, elevated invasion ability and cellular quiescence.

Mechanistically, both SLUG and GAS1 were identified and further demonstrated to be direct targets of NICD4. Interestingly, the binding sites for SLUG identified in this study are just the same as those reported by Niessen K. et al, supporting that SLUG is a genuine direct target of NOTCH4 not only in heart development but also in TNBC ML-BCSC maintenance and survival 42. Besides, GAS1 was identified as a novel NOTCH4 target gene in this work to contribute to cancer stemness, although GAS1 has long been regarded as a tumor suppressor gene due to its canonical role in inhibiting cell proliferation 48. Noteworthily, evidence emerges recently to support a role of GAS1 in regulating stem cells 47 as well as CSCs, mainly contributed to hedgehog pathway activation 51. Otherwise, we also observed a pro-apoptotic role of GAS1 when it was overexpressed alone in TNBC cells, which could be alleviated by SLUG. These results depicted such a scenario, where the higher NOTCH4 activity in TNBC cells harnessed the previously regarded tumor suppressor GAS1 to serve as an accomplice in tumor progression 36, both as a cell cycle arrester and a mediator to hedgehog signaling, in maintaining the ML-BCSC status.

The above results suggested that the NOTCH4-SLUG-GAS1 circuit might serve as a promising target to treat TNBC. Our results confirmed this conception, as interference of either SLUG or GAS1 significantly abrogated the malignant traits of TNBC cells. However, the current widely used γ-Secretase inhibitors (GSI), such as MK-0752 and RO4929097, although efficient in inhibiting the activation of NOTCH1-3, were shown to be inefficient in inhibiting NOTCH4 activation 20, 24 and showed minimal clinical activity 52. In fact, there is emerging evidence showing that NOTCH pathway can also be activated in non-canonical manners where ligand binding as well as γ-Secretase is not required 53, 54. Hopefully, there are more inhibitors being developed in laboratory, which provide the possibility to target NOTCH4 activation 55, 56. Another possible reason for the higher NICD4 in TNBC was attributed to the disrupted pathways targeting NICD4 for degradation under normal circumstances 53, 57. In addition, we unexpectedly observed a tumor suppressor function of NOTCH4 in the non-TNBC cell line MCF-7, where forced activation of NOTCH4 induced cell differentiation and inhibited invasion. Indeed, seemingly controversial results have been previously reported in melanoma to state that NOTCH4 functions as either a cancer stem cell marker 25 or a tumor-suppressor gene 58. These results reflect the cell-context dependent roles of NOTCH4 and highlight the significance of targeting NOTCH4 signaling in defined subtypes to achieve a precision therapy.

In conclusion, our work demonstrated that NOTCH4 might supplant CD24^-^CD44^+^ in labeling TNBC stem cells for its accuracy and revealed the mechanisms by which NOTCH4 regulated stemness, chemoresistance and invasion of the TNBC cells. NOTCH4 drove ML-BCSCs into a quiescent state to acquire chemoresistance through GAS1, and induced EMT via upregulating SLUG to increase invasion and metastasis. In the meanwhile, SLUG also served to eliminate the pro-apoptotic side-effect of GAS1. Hence, NOTCH4-SLUG-GAS1 composed up a circuit to promote ML-BCSC maintenance by simultaneously inducing EMT and cellular quiescence, which could be a potential therapeutic target to inhibit metastasis and chemoresistance of TNBC.

## Abbreviations

ALDH: aldehyde dehydrogenase
BCSC: breast cancer stem cell
DMFS: distant-metastasis free survival
EMT: epithelial-to-mesenchymal transition
ER: estrogen receptor
GSIs: γ-secretase inhibitors, H-Score, histochemical staining-score
IHC: immunohistochemistry
ML-BCSC: mesenchymal-like breast cancer stem cell
NICD4: NOTCH4 intracellular domain
OS: overall survival
RFS: relapse free survival
TCGA: The Cancer Genome Atlas
TNBC: triple-negative breast cancer
