# Supplementary material for: NOTCH4 maintains quiescent mesenchymal-like breast cancer stem cells via transcriptionally activating SLUG and GAS1 in triple-negative breast cancer
Source: Theranostics. 2020 Jan 19;10(5):2405–21. doi: 10.7150/thno.38875 (PMC7019177; doi:10.7150/thno.38875)
Supplement: Supplementary file 1 — Supplementary figures and tables. [file thnov10p2405s1.pdf]

Supplementary Figures and Legends

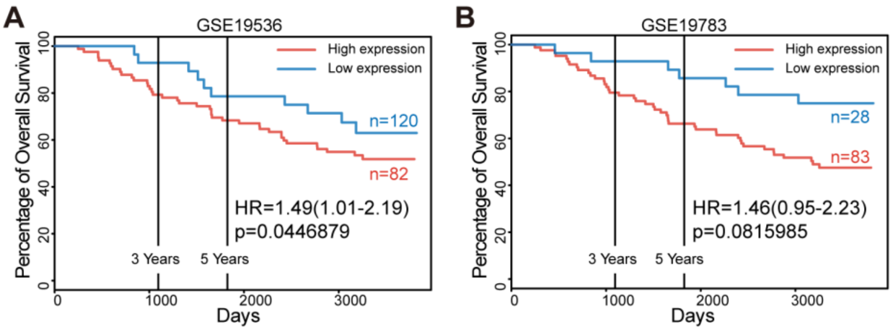

Figure S1. Higher NOTCH4 expression predicted poorer overall survival in breast cancer. (A-B) Overall survival was analyzed at PROGgene-v2 website (http://genomics.jefferson.edu/proggene/) of two independent breast cancer datasets as indicated. Expression level was automatically divided by the Online tool. Datasets, Hazard Ratio (HR) and p-value were indicated.

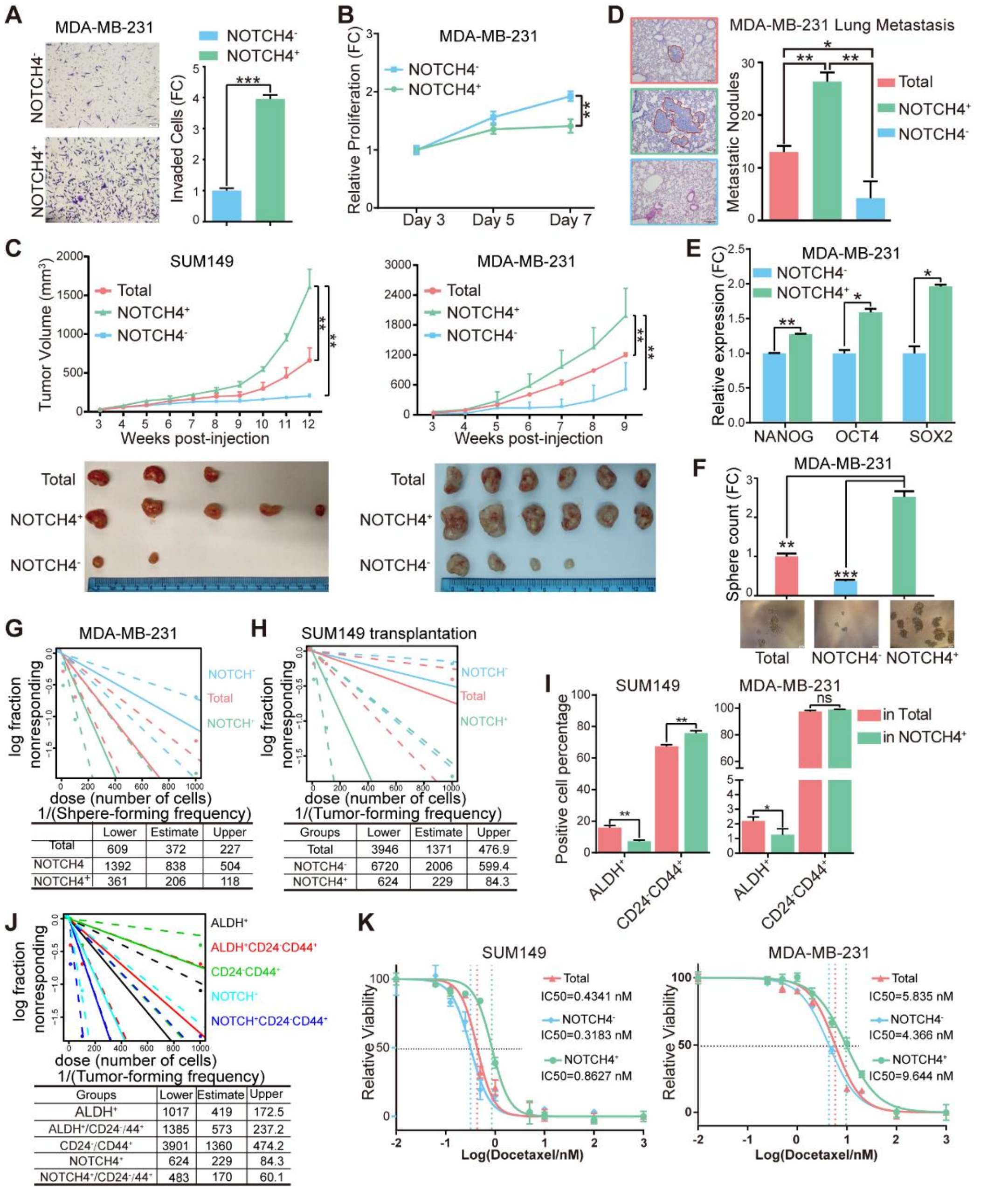

Figure S2. NOTCH4$^+$ subpopulation enriched BCSCs. (A-H) Sorted cells according to NOTCH4 expression were used to assess: **(A)** invasion ability by transwell assay; **(B)** proliferation ability via MTT assay; (**C**) tumorigenesis using in vivo transplantation (n=6); (**D**) metastatic nodules formed by sorted MDA-MB-231 cells in lungs; **(E)** stemness regulating transcription factors by qRT-PCR; **(F)** mammosphere formation ability; **(G)** BCSC frequency by serial dilution mammosphere formation; **(H)** BCSC frequency by serial dilution tumor transplantation; For statistics, data was shown as Mean±SD of fold change. **(I)** Overlapping between NOTCH4$^+$ and ALDH$^+$ or CD24$^-$CD44$^+$ populations, proportion of NOTCH4$^+$ cells in total population or in defined

populations are shown as Mean±SD; **(J)** BCSC frequency comparison between different populations labeled by indicated markers was performed by in vivo serial dilution transplantation assay. **(K)** Chemosensitivity of different cell subpopulations to docetaxel was determined via MTT. * P<0.05, ** P<0.01, *** P<0.001.

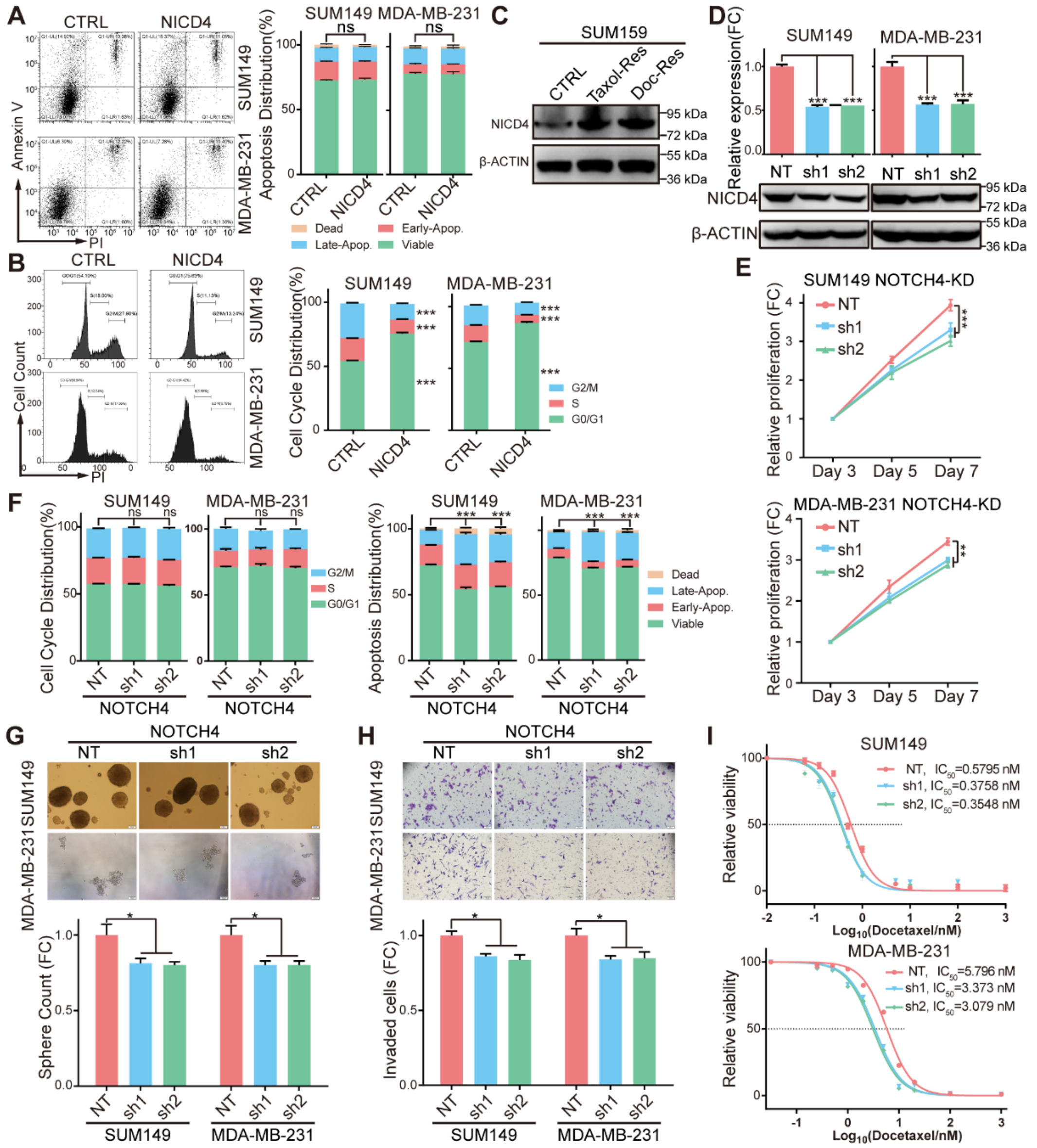

Figure S3. NOTCH4 was vital for TNBC cell survival. (A-B) Apoptosis **(A)** and cell cycle distribution **(B)** were determined by flow cytometry in NICD4 overexpressing SUM149 and MDA-MB-231, with all constituents indicated in the related bar graphs; **(C)** NICD4 expression in two taxane-resistant cell strains derived from SUM159 was determined by western blotting, with β-ACTIN used as loading control; **(D)** NOTCH4 was stably knocked down by shRNA and the efficiency was determined by qRT-PCR with TBP as internal control and western blotting with β-ACTIN as loading control, respectively. **(E-I)** Subsequently, the following characteristics were determined in the NOTCH4-knockdown cells: **(E)** proliferation ability via MTT (left panel) and cell cycle distribution by flow cytometry (right panel), **(F)** apoptosis distribution by flow cytometry (Early-Apop.=early apoptosis, Late-Apop.=late apoptosis), **(G)** mammosphere formation ability, **(H)** invasion ability via transwell assay, and **(I)** chemoresistance ability via MTT assay. For statistics, data was shown as Mean±SD of

fold change. Triple independent experiments were carried out and representative results were represented. ns=not significant, * $P<0.05$, ** $P<0.01$, *** $P<0.001$.

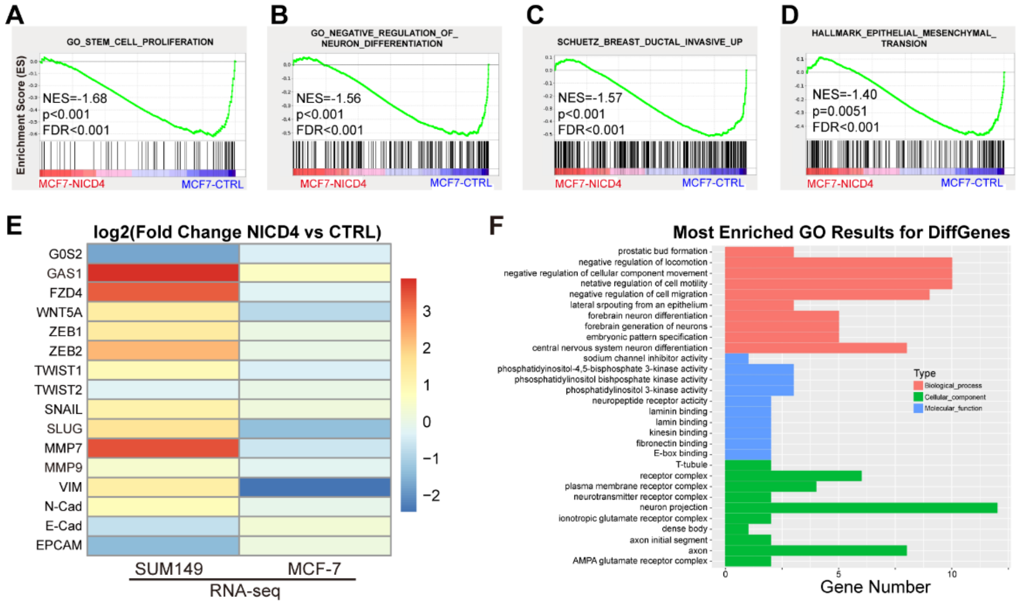

Figure S4. Activated NOTCH4 (NICD4) failed to induce an ML-BCSC expression profile in non-TNBC cell MCF-7. **(A-D)** GSEA analysis of RNA-seq data of NICD4 overexpressing MCF-7 exhibited enrichment of **(A)** stemness regulating genes, **(B)** neuron-like differentiation associated genes, **(C)** invasion enhancing genes and **(D)** epithelial-to-mesenchymal transition associated genes. **(E)** Fold change of selected genes was shown as heatmap according to the RNA-seq results of SUM149 and MCF-7. Data was shown as $\log_2$ of fold change relative to the control group of each cell line. **(F)** GO analysis of the MCF-7 RNA-seq data indicated enriched negative regulators of motility associated genes and differentiation related genes.

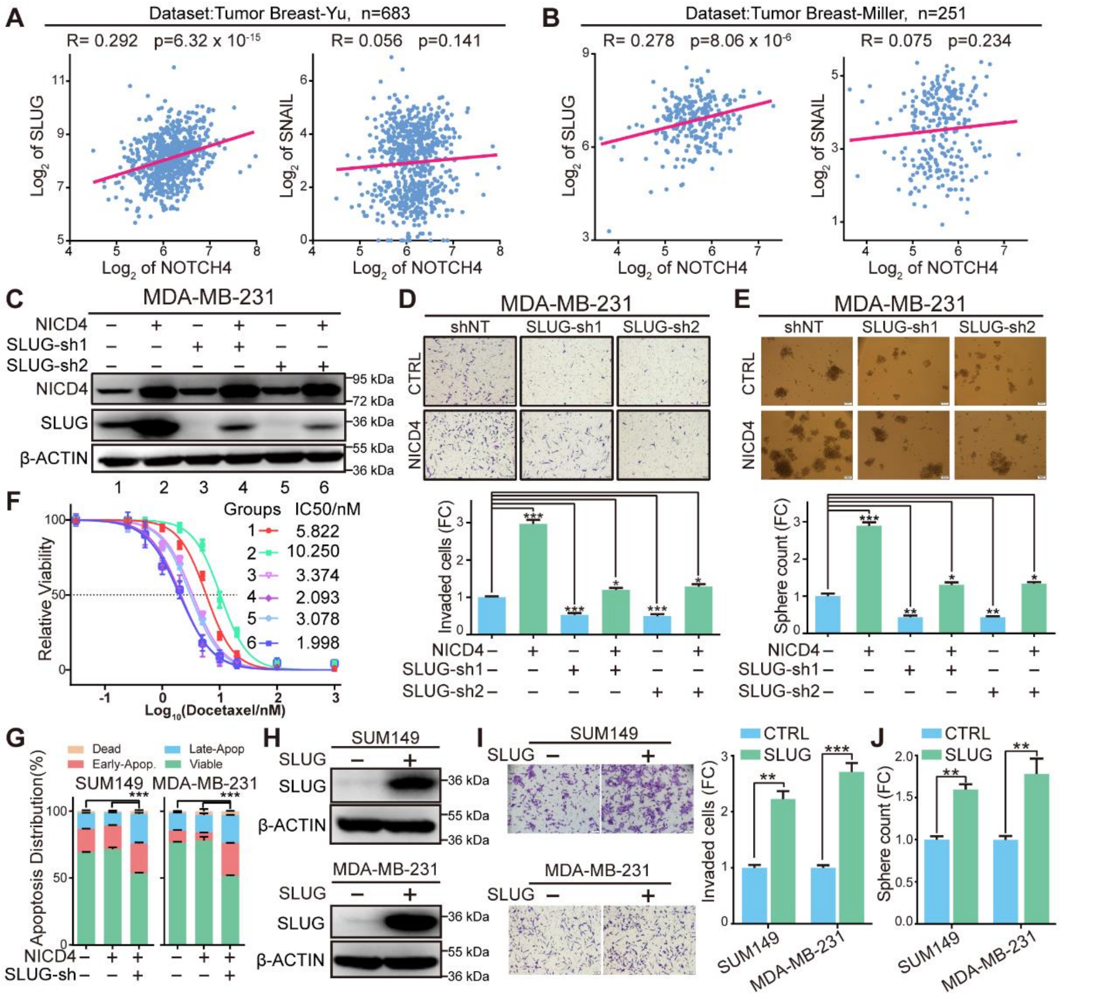

Figure S5. SLUG mediated the NOTCH4-induced invasion ability. **(A-B)** Expression correlation between NOTCH4 and SLUG or SNAIL were plotted in two different datasets; **(C)** SLUG was knocked down in NICD4-overexpressing MDA-MB-231 and determined by western blotting. **(D-G)** We then determined **(D)** invasion via transwell assay, **(E)** mammosphere formation, **(F)** chemoresistance ability to Docetaxel via MTT and **(G)** apoptosis distribution by flow cytometry (Early-Apop., early apoptosis; Late-Apop., late apoptosis). **(H)** SLUG was overexpressed in SUM149 and MDA-MB-231, which was confirmed by western blotting; **(I-J)** We then determined **(I)** invasion via transwell assay and **(J)** mammosphere formation ability in the overexpressing cells. For statistics, data was shown as Mean±SD of fold change. Triple independent experiments were carried out and representative results were represented. In western blotting, β-ACTIN was used as loading control. * P<0.05, ** P<0.01, *** P<0.001.

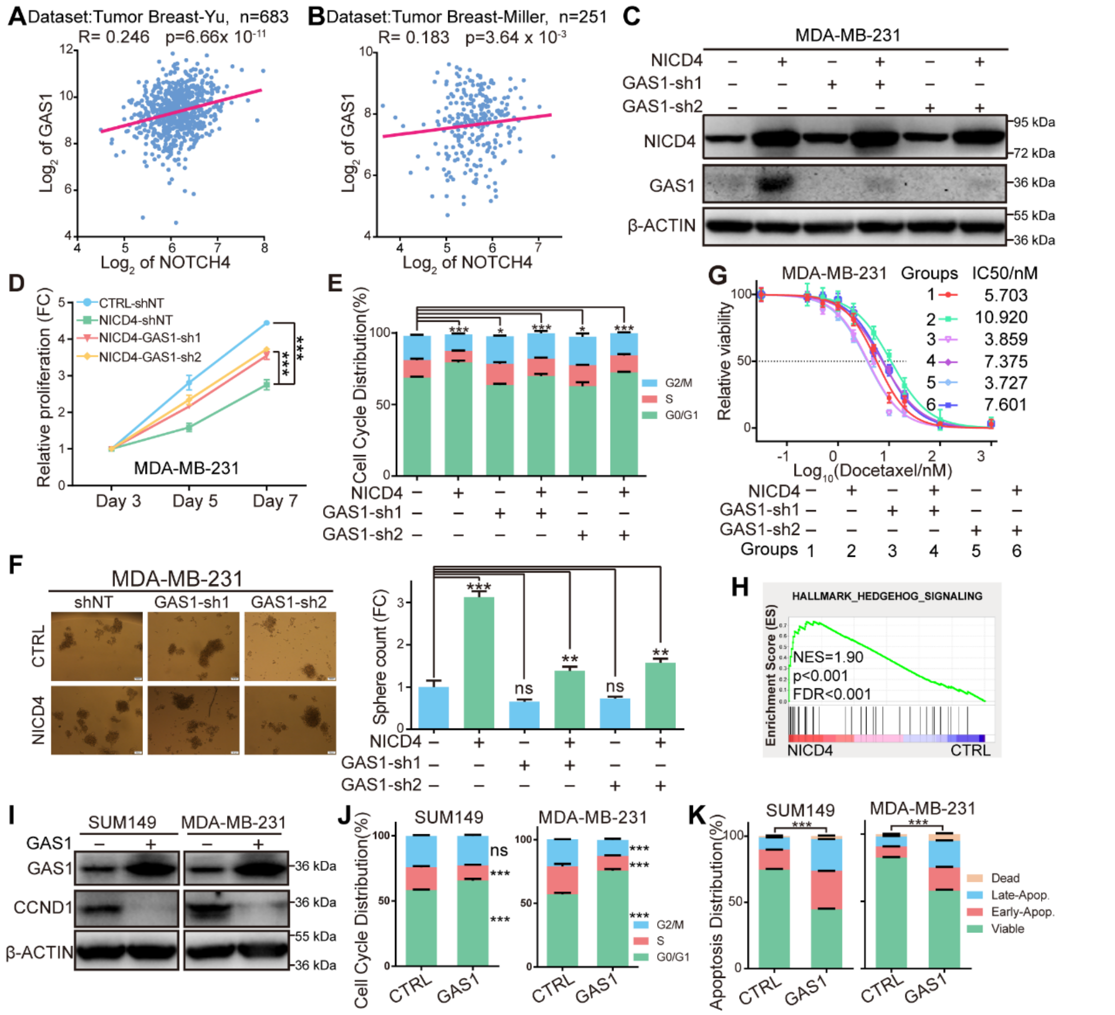

Figure S6. GAS1 induces quiescence in NICD4-overexpressing cells. (A-B) Expression correlation between NOTCH4 and GAS1 was plotted using two independent datasets. **(C)** GAS1 was knocked down in NICD4-overexpressing MDA-MB-231 and then confirmed by western blotting. **(D-G)** Subsequently, we determined **(D)** proliferation via MTT, **(E)** cell cycle distribution via flow cytometry, **(F)** mammosphere formation, and **(G)** chemoresistance ability by MTT. **(H)** Enriched hedgehog pathway was identified in GSEA analysis of NICD4-everexpressing SUM149. **(I)** GAS1 was overexpressed in SUM149 and MDA-MB-231, and GAS1 and CCND1 were determined by western blotting. **(J-K)** We then determined **(J)** cell cycle and **(K)** apoptosis in the GAS1-overexpressing cells (Early-Apop., early apoptosis; Late-Apop., late apoptosis). For statistics, data was shown as Mean±SD of fold change. Triple independent experiments were carried out and representative results were represented. β-ACTIN was used as loading control in western blotting. ns=not significant, ** P<0.01, *** P<0.001.

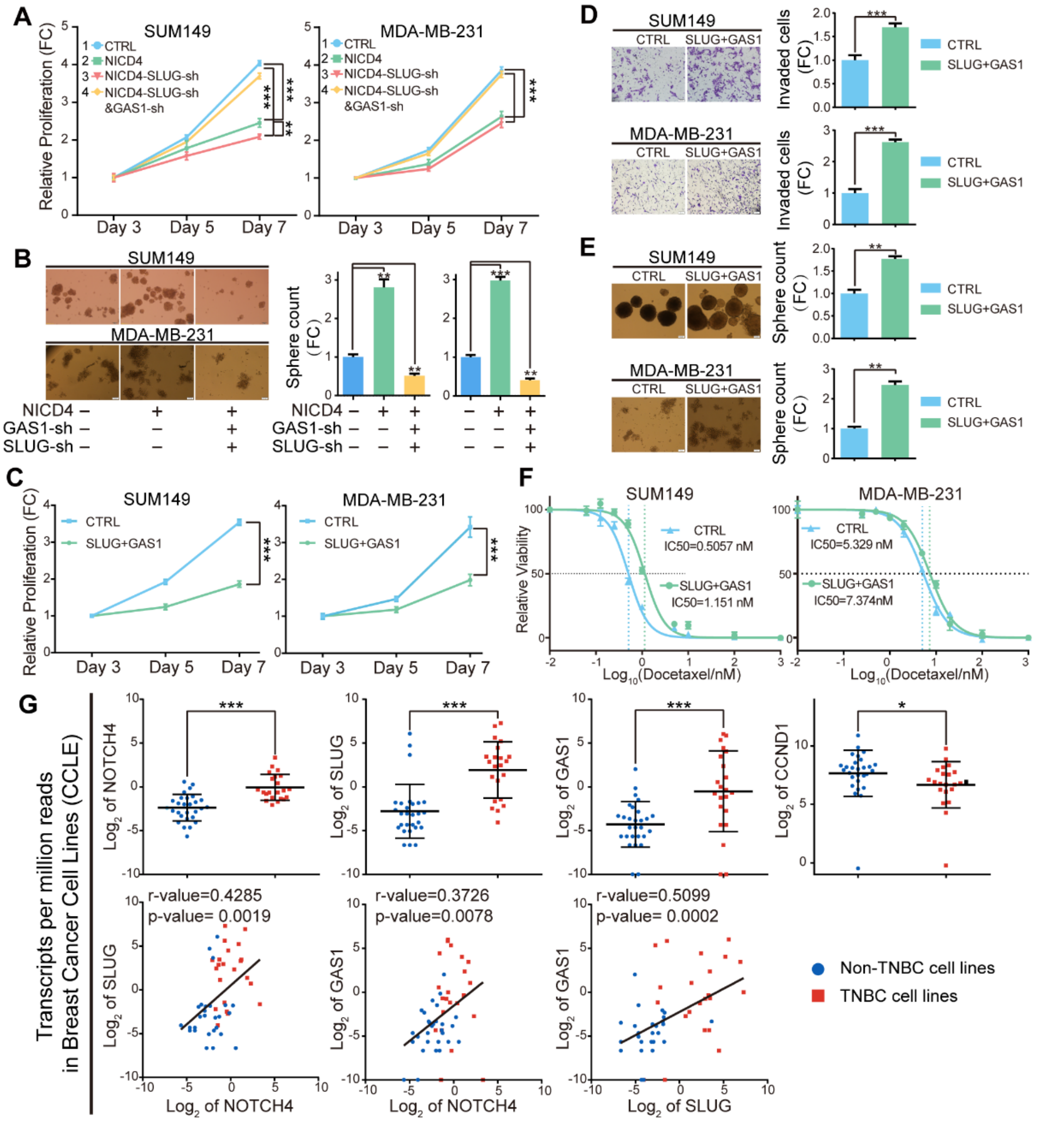

Figure S7. SLUG and GAS1 mediated the effects of NOTCH4. (A-B) Knockdown of SLUG and GAS1 in NICD4 overexpressing cells withdrew the effects of NICD4 in promoting proliferation (**A**) and enhancing mammosphere formation (**B**); **(C-F)** We overexpressed SLUG and GAS1 in SUM149 and MDA-MB-231 and then determined the following functional effects: **(C)** proliferation ability by MTT assay, **(D)** invasion ability by transwell assay, **(E)** mammosphere formation, and **(F)** chemoresistance ability via MTT assay. Triple independent experiments were carried out and representative results were represented; **(G)** RNA-seq data was retrieved from CCLE and the transcripts per million reads (TPM) of NOTCH4, SLUG, GAS1 and CCND1 were analyzed (upper panel) and correlation between them were plotted (lower panel); TNBC cells were plotted in red squares and non-TNBC cells in blue circles. * P<0.05, ** P<0.01, *** P<0.001.

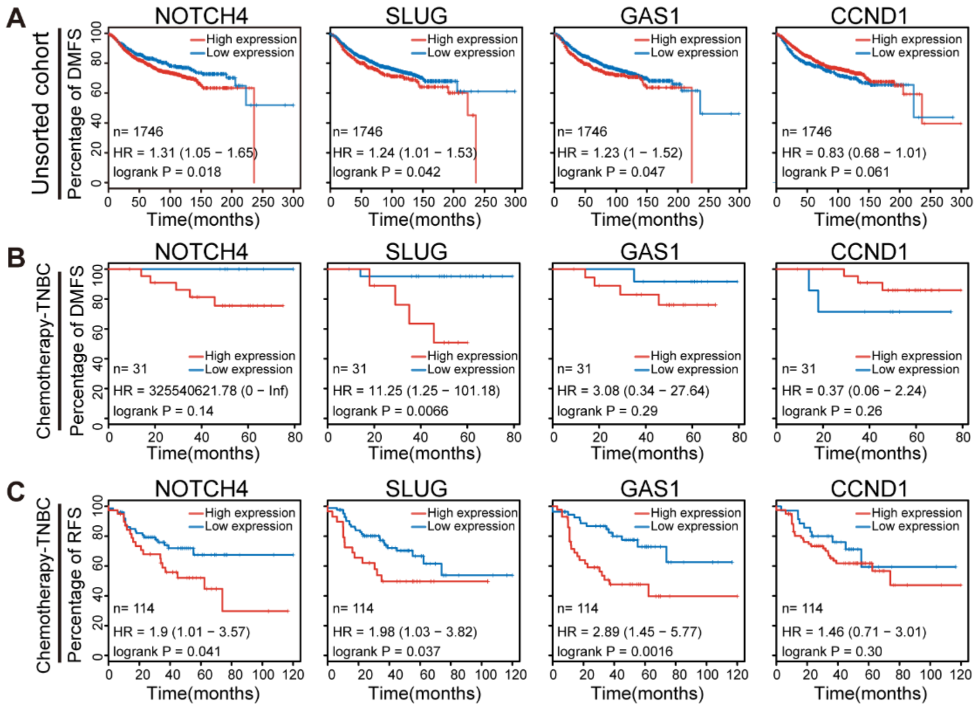

Figure S8. NOTCH4, SLUG and GAS1 predicted poor DMFS and RFS. (A) DMFS was analyzed using all breast cancer datasets at Kaplan Meier (https://kmplot.com/analysis/) for NOTCH4, SLUG, GAS1 and CCND1. **(B)** DMFS and **(C)** RFS were analyzed for NOTCH4, SLUG, GAS1 and CCND1 in chemotherapy-received patients, with Hazard Ratio (HR) and p-value indicated. Expression level was automatically divided by Kaplan Meier.

Supplementary Tables:

Table S1 Primers used to construct overexpression vectors

Vector	Forward primer (5' to 3')	Reverse primer (5' to 3')
pSIN-EF1α-NICD4	TGTCGTGAGGAATTGGGATCCGCCACCATGGTCCTCCAGCT	GCTTCATATGTTCGAAGAATTCCTATTTTTTACCCTCTCCT
pSIN-EF1α-SLUG	GTCGTGAGGAATTGGGATCCGCCACCATGCCGCGCTCCTTCCTGGT	ATATGTTCGAAGAATTCTCATCAGTGTGCTACACAGCAGC
pSIN-EF1α-GAS1	TGTCGTGAGGAATTGGGATCCGCCACCATGGTGGCCGCGCTGCT	CTTCATATGTTCGAAGAATTCTCAAAAGAGCGGCCCAAGCAG

Table S2 Primers used to construct shRNA vectors

shRNA clone	Forward primer (5' to 3')<br>Reverse primer (5' to 3')
Scramble shRNA	CTCGAGTTGGTGCTCTTCATCTTGTTGTTTTTGAATTCTCGACCTCGAG<br>CCAACTCGAGTTGGTGCTCTTCATCTTGTTGCGGTGTTTCGTCCTTTCC
NOTCH4-sh1	CTCGAGATTCTGACATTGGTGGCTGACTTTTTGAATTCTCGACCTCGAG<br>GAATCTCGAGATTCTGACATTGGTGGCTGACCGGTGTTTCGTCCTTTCC
NOTCH4-sh2	CTCGAGTGAGCAGTTCTGTCCATCGTATTTTTGAATTCTCGACCTCGAG<br>CTCACTCGAGTGAGCAGTTCTGTCCATCGTACGGTGTTTCGTCCTTTCC
SLUG-sh1	CTCGAGTAACTCTCATAGAGATACGGGTTTTTGAATTCTCGACCTCGAG<br>TTTACTCGAGTAACTCTCATAGAGATACGGGCGGTGTTTCGTCCTTTCC
SLUG-sh2	CTCGAGTTTAAGGCACCTGAGTTCGCGTTTTTGAATTCTCGACCTCGAG<br>AAATCTCGAGTTTAAGGCACCTGAGTTCGCGCGGTGTTTCGTCCTTTCC
GAS1-sh1	CTCGAGAAATATGCTAATAGACAGCCCTTTTTGAATTCTCGACCTCGAG<br>ATTTCTCGAGAAATATGCTAATAGACAGCCCCGGTGTTTCGTCCTTTCC
GAS1-sh2	CTCGAGAAAGAGACTTTCATACATGGCTTTTTGAATTCTCGACCTCGAG<br>CTTTCTCGAGAAAGAGACTTTCATACATGGCCGGTGTTTCGTCCTTTCC

Note: The guide sequences of shRNA were highlighted in red letter, detailed information of primer design for shRNA construction can be found in our previous work [1].

Table S3 qRT-PCR primers used in this study

Genes	Forward primer (5' to 3')	Reverse primer (5' to 3')
NOTCH1	TGATCCTGACTGCGATGAGAG	CTTGTCTGTTCTTCTGACCCC
NOTCH2	AAAAATGGGGCCAACCGAGAC	TTCATCCAGAAGGCGCACAA
NOTCH3	CGTGGCTTCTTTCTACTGTGC	CGTTCACCGGATTTGTGTCAC
NOTCH4	GCGGAGGCAGGGTCTCAACGGATG	AGGAGGCGGGATCGGAATGT
NANOG	AATACCTCAGCCTCCAGCAGATG	TGCGTCACACCATTGCTATTCTTC
OCT4	CTGGGTTGATCCTCGGACCT	CACAGAACTCATACGGCGGG
SOX2	GCACATGAACGGCTGGAGCAACG	TGCTGCGAGTAGGACATGCTGTAGG
G0S2	CCAAGGAGATGATGGCCCAG	GCTGCACACAGTCTCCATCA
EpCAM	TGATCCTGACTGCGATGAGAG	CTTGTCTGTTCTTCTGACCCC
GAS1	ATGCCGCACCGTCATTGAG	TCATCGTAGTAGTCGTCCAGG
FZD4	GTCTTTCAGTCAAGAGACGCTG	GTTGTGGTCGTTCTGTGGTG
WNT5A	GCCAGTATCAATTCCGACATCG	TCACCGCGTATGTGAAGGC
ZEB1	TTACACCTTTGCATACAGAACCC	TTTACGATTACACCCAGACTGC
ZEB2	CGGTGCAAGAGGCGCAAACA	GGAGGACTCATGGTTGGGCA
TWIST1	CACGAGCGGCTCAGCTACGC	AATGACATCTAGGTCTCCGGCCC
TWIST2	CAAGCTGAGCAAGATCCAGAC	GGTCATCTTATTGTCCATCTCG
SNAIL	TCGGAAGCCTAACTACAGCGA	AGATGAGCATTGGCAGCGAG
SLUG	CTGGGCGCCCTGAACATGCAT	GCTTCTCCCCCGTGTGAGTTCTA
MMP7	GTGGTCACCTACAGGATCGTA	CTGAAGTTTCTATTTCTTTCTTGA
MMP9	AGACCTGGGCAGATTCCAAAC	CGGCAAGTCTTCCGAGTAGT
VIM	GACGCCATCAACACCGAGTT	CTTTGTCGTTGGTTAGCTGGT
N-Cad	CACCCTGGCTTTGACGCCGA	AAAATTCACTCTGCCCAGGACGCG
E-Cad	CGCCATCCAGACCGACCCAA	GTCGATTGGTTTGACCACGGTGAC
TBP	TGCACAGGAGCCAAGAGTGAA	CACATCACAGCTCCCCACCA

Table S4 qRT-PCR primers used for ChIP DNA quantification

Primers	Forward primer (5' to 3')	Reverse primer (5' to 3')
SLUG-site1	TAGCACCACATAAAAGCAGGGG	CACCGGACATTCTCTCACACT
SLUG-site2	AGCCATGGCGATATGTGTTTTC	TGTTCGGATGTAGGCACCTG
SLUG-site3	TCCACGCAAAGTAAAGAAACCC	TTCTCCCGGAGCCAGTTTTC
GAS1-site1	GGTCCGCCTTACCTCAGTCT	CACTAGGAGATTCCTCCGGC
GAS1-cluster1	ATGCGAGTCTGAAGGTGGTC	AAGATGAGATTTGGGTGGG

Table S5 Primers used to construct luciferase promoter reporters and mutants

Vector	Forward primer (5’ to 3’)	Reverse primer (5’ to 3’)
SLUG-pro-WT	GAGGAACTTGGTTAGGTACCCCCCCTCCAGAGTGATCATACC	TGGAAGCCATGGTGGCTAGCCTTGCCAGCGGGTCTGG
SLUG-pro-Site-1 mutation	TAAAAAAAAAGATGCACTGTAATACATG	TGCATCTTTTTTTTTAACCCTTAAAGGGTTGTATGGGTCTTTAGAG
SLUG-pro-Site-2 mutation	AAAATGTGTGTTTTGACCCTTATGGAGTGAAAAGCAAGGAGGACTCCTGCT	CAAAACACACATTTTTGTTACAGATATAGCACAGTTGAG
SLUG-pro-Site-3 mutation	AACCTCACGGATCTAATTTATTCC	TAGATCCGTGAGGTTACCCTTCGCACCTTGGGTTTCTTTACTT
GAS1-pro-WT	GAGGAACTTGGTTAGGTACCGGTCAATGCCTCCTTACATTTTGCACC	TGGAAGCCATGGTGGCTAGCAAGTTTGTCCAAGTCCTGCC
GAS1-pro-Site-1 mutation	ACGGCAGACCAAGCGCCTCT	CGCTTGGTCTGCCGTCCCTTGCTGCACCTCTGCAGTCC
GAS1-pro-Site-2 mutation	CATGGGGGTGTATCTAAGGGTTGCTGTTTTCATGACAGTGAATG	AGATACACCCCCATGATTCAG
GAS1-pro-Site-3 mutation	TTGTAGCTCCCATAAAAGGGTCATATTGTGGGAGGGACC	TTATGGGAGCTACAAGATGAGATTTG
GAS1-pro-Site-4 mutation	AGGTTTGGCTCTGTGAAGGGTCCCAAATCTCATCTTGTAGCT	CACAGAGCCAAACCTTATCAG

Table S6 Antibodies used in this study

Genes	Suppliers	Category Number	Dilution
NOTCH4	CST	#2423S	1:1000
SLUG	CST	#9585S	1:1000
SNAIL	CST	#3578S	1:1000
Vimentin	CST	#5741	1:1000
E-Cadherin	CST	#144725	1:1000
GAS1	GeneTex	#GTX101732	1:1000
β-ACTIN	TransGen	#HC201	1:2000

Reference:

1. Zhou L, Sheng D, Deng Q, Wang D, Liu S. Development of a novel method for rapid cloning of shRNA vectors, which successfully knocked down CD44 in mesenchymal triple-negative breast cancer cells. Cancer Commun (Lond). 2018; 38: 57.
